# Primary cilia contribute to the aggressiveness of atypical teratoid/rhabdoid tumors

**DOI:** 10.1038/s41419-022-05243-4

**Published:** 2022-09-20

**Authors:** Lena Blümel, Nan Qin, Johannes Berlandi, Eunice Paisana, Rita Cascão, Carlos Custódia, David Pauck, Daniel Picard, Maike Langini, Kai Stühler, Frauke-Dorothee Meyer, Sarah Göbbels, Bastian Malzkorn, Max C. Liebau, João T. Barata, Astrid Jeibmann, Kornelius Kerl, Serap Erkek, Marcel Kool, Stefan M. Pfister, Pascal D. Johann, Michael C. Frühwald, Arndt Borkhardt, Guido Reifenberger, Claudia C. Faria, Ute Fischer, Martin Hasselblatt, Jasmin Bartl, Marc Remke

**Affiliations:** 1 Department of Pediatric Oncology, Hematology and Clinical Immunology, Heinrich Heine University Düsseldorf, Medical Faculty, and University Hospital Düsseldorf, Düsseldorf, Germany; 2 German Cancer Consortium (DKTK), partner site Essen/Düsseldorf, Düsseldorf, Germany; 3 Institute of Neuropathology, Heinrich Heine University Düsseldorf, Medical Faculty, and University Hospital Düsseldorf, Düsseldorf, Germany; 4 Institute of Neuropathology, University Hospital Münster, Münster, Germany; 5 Instituto de Medicina Molecular João Lobo Antunes, Faculdade de Medicina da Universidade de Lisboa, Lisbon, Portugal; 6 Molecular Proteomics Laboratory, Biological and Medical Research Center (BMFZ), Heinrich Heine University Düsseldorf, Düsseldorf, Germany; 7 Institute of Molecular Medicine, Heinrich Heine University Düsseldorf, Medical Faculty, and University Hospital Düsseldorf, Düsseldorf, Germany; 8 Department of Pediatrics, Center for Family Health, Center for Rare Diseases and Center for Molecular Medicine, University Hospital Cologne and Faculty of Medicine, University of Cologne, Cologne, Germany; 9 Department II of Internal Medicine, University Hospital Cologne and Faculty of Medicine, University of Cologne, Cologne, Germany; 10 Department of Pediatric Hematology and Oncology, University Hospital Münster, Münster, Germany; 11 Hopp Childrens Cancer Center (KiTZ), Heidelberg, Germany; 12 Division of Pediatric Neurooncology, German Cancer Research Center (DKFZ) and German Cancer Consortium (DKTK), Heidelberg, Germany; 13 Princess Máxima Center for Pediatric Oncology, Utrecht, the Netherlands; 14 Department of Hematology and Oncology, Heidelberg University Hospital, Heidelberg, Germany; 15 Swabian Childrens´ Cancer Center, Pediatrics and Adolescent Medicine, University Medical Center, Augsburg, Germany; 16 Neurosurgery Department, Hospital de Santa Maria, Centro Hospitalar Universitário Lisboa Norte (CHULN), Lisbon, Portugal; 17 Group for Interdisciplinary Neurobiology and Immunology - INI-research, Institute of Zoology, University of Hamburg, Hamburg, Germany

**Keywords:** CNS cancer, Oncogenesis

## Abstract

Atypical teratoid/rhabdoid tumor (AT/RT) is a highly malignant brain tumor in infants that is characterized by loss of nuclear expression of SMARCB1 or SMARCA4 proteins. Recent studies show that AT/RTs comprise three molecular subgroups, namely AT/RT-TYR, AT/RT-MYC and AT/RT-SHH. The subgroups show distinct expression patterns of genes involved in ciliogenesis, however, little is known about the functional roles of primary cilia in the biology of AT/RT. Here, we show that primary cilia are present across all AT/RT subgroups with specific enrichment in AT/RT-TYR patient samples. Furthermore, we demonstrate that primary ciliogenesis contributes to AT/RT biology in vitro and in vivo. Specifically, we observed a significant decrease in proliferation and clonogenicity following disruption of primary ciliogenesis in AT/RT cell line models. Additionally, apoptosis was significantly increased via the induction of STAT1 and DR5 signaling, as detected by proteogenomic profiling. In a *Drosophila* model of *SMARCB1* deficiency, concomitant knockdown of several cilia-associated genes resulted in a substantial shift of the lethal phenotype with more than 20% of flies reaching adulthood. We also found significantly extended survival in an orthotopic xenograft mouse model of AT/RT upon disruption of primary ciliogenesis. Taken together, our findings indicate that primary ciliogenesis or its downstream signaling contributes to the aggressiveness of AT/RT and, therefore, may constitute a novel therapeutic target.

## Introduction

Atypical teratoid/rhabdoid tumors (AT/RTs) are rare but highly malignant brain tumors primarily diagnosed in infants [1]. They are characterized by their fast growth and a short history of progressive symptoms, often leading to intracranial hypertension requiring urgent intervention [2]. Bi-allelic mutations of SWI/SNF chromatin remodeling complex member *SMARCB1* (also known as *hSNF5/INI1*) causing loss of nuclear SMARCB1 protein expression are the characteristic genetic lesion in these tumors [3]. In rare cases, in which mutations in *SMARCB1* are absent, tumors harbor mutations in *SMARCA4*, which is also part of the SWI/SNF complex [4, 5]. For decades, the mainstay of care has been based on a multimodal approach consisting of maximal surgical resection, (high-dose) chemotherapy with or without autologous stem cell transplantation and cranio-spinal irradiation [6, 7]. Despite intensification of the therapeutic approaches, however, up to 50% of patients with AT/RT demonstrate early disease progression or relapse [8]. Furthermore, therapy escalation is associated with an increased risk for severe side effects and long-term sequelae, affecting neurologic, endocrine and cognitive functions, thus, severely affecting quality of life in these young patients [9]. Therefore, a better understanding of the biology of AT/RT is urgently needed to identify novel, target-directed treatment strategies.

Recent large-scale multicenter studies [10–12] have significantly advanced our understanding of the molecular pathogenesis of these tumors, thereby uncovering three molecular subgroups of AT/RT (AT/RT-TYR, AT/RT-MYC and AT/RT-SHH) [11]. Although the AT/RT genome has been shown to be remarkably homogeneous across subgroups with no additional recurrent mutations identified except for the prototypic *SMARCB1* (or *SMARCA4*) mutations, epigenetic differences across subgroups have been shown to be striking, including different methylation profiles and enhancer activities. Moreover, highly distinct gene expression profiles form the basis for the subgroup designations. While MYC is highly expressed in the MYC group of AT/RT, the sonic hedgehog (SHH) group is characterized by active SHH signaling, while the term TYR of the TYR group is derived from tyrosinase, one of several highly expressed melanosomal markers in this subgroup. Interestingly, it has been shown that also genes involved in ciliogenesis are expressed in AT/RT, with highest levels detected in the TYR subgroup [11].

Primary cilia are microtubuli-based antenna-like structures that are anchored to the cell by the basal body and arise from the surface of virtually every cell in the human body [13]. By now, they are known to be crucial regulators of cell signaling. Most prominently, primary cilia were identified as an essential mediator of the SHH pathway [14]. Given the important role for primary cilia in cell signaling, it is not surprising that a dysfunction of primary cilia is frequently associated with pathologies [15]. Originally, primary cilia were found to be involved in a variety of diseases commonly referred to as ciliopathies [16], but growing evidence suggests that primary cilia also play a pivotal role in different types of cancers [17], including skin [18], epithelial [19–21], and also brain cancers [22–24]. Notably, the way primary cilia regulate tumorigenesis seems to differ between tumor types and within tumor subtypes. We therefore aimed at investigating the role of primary cilia in AT/RT.

Here we show a subgroup-specific expression of primary cilia in AT/RT patient samples using immunofluorescence. Furthermore, we demonstrate that disruption of primary ciliogenesis decreases proliferation and clonogenicity, and increases apoptosis in AT/RT cell line models. By using a *Drosophila* model of *SMARCB1* deficiency and an orthotopic xenograft mouse model of AT/RT, we confirm that disruption of primary ciliogenesis provides a significant survival benefit, emphasizing the functional relevance of this organelle conserved across species with *SMARCB1* deficiency. Notably, proteogenomic profiling reveals that primary ciliogenesis drives signal transducer and activator of transcription (STAT1) and death receptor 5 (DR5) signaling in AT/RT cell line models. Altogether, these results indicate that primary cilia contribute to the aggressiveness in these malignant embryonal brain tumors with dismal prognosis.

## Results

### Detection of primary cilia in AT/RT patient samples and cell lines

**Fig. 1:**
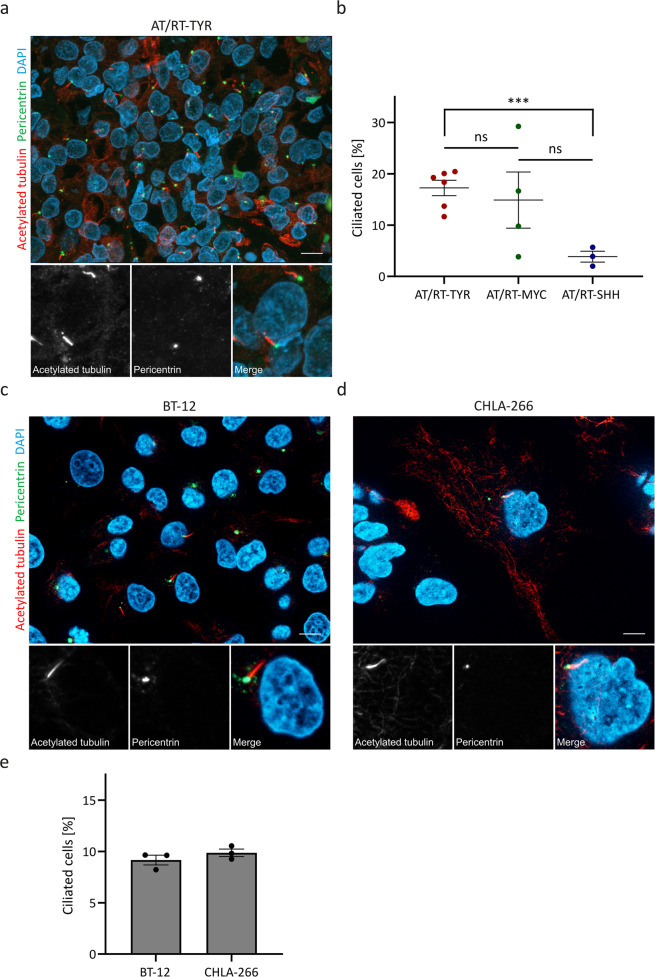
Detection of primary cilia in AT/RT tumor tissue sections and cell lines. Tumor tissue sections of AT/RT patients were deparaffined and stained for pericentrin (green) and acetylated tubulin (red) to detect the basal body and the axoneme of the primary cilium, respectively. Nuclei were counterstained with 4´,6‐diamidino‐2‐phenylindole (DAPI, blue). Overall, a total of 13 different AT/RT tumor tissue samples were examined, including six AT/RT‐TYR, four AT/RT‐MYC and three AT/RT‐SHH tumors. A representative immunofluorescence image for an AT/RT‐TYR tumor is shown in (**a**). **b** Quantification of the percentage of ciliated cells in each of the AT/RT tumor tissue samples revealed a significant difference across tumor subgroups. AT/RT cell lines BT‐12 (**c**) and CHLA‐266 (**d**) were fixed and stained using the same immunofluorescence protocol used for the AT/RT tumor tissue samples. Quantification of the percentage of ciliated cells in BT-12 and CHLA-266 cells is shown in (**e**). ns: not significant; ***p < 0.001 (*t*-test). The scale bar corresponds to 10 μm.

In this study, we examined tumor tissue sections from 13 AT/RT patients. Overall, these samples included six AT/RT-TYR, four AT/RT-MYC and three AT/RT-SHH tumors (Supplementary Table 1). We performed immunofluorescence to detect primary cilia in these samples. To this aim, we employed antibodies directed against pericentrin and acetylated tubulin that detect the basal body and the axoneme of the primary cilium, respectively. We detected primary cilia in all tumor tissue sections of the AT/RT patients examined (a representative immunofluorescence image for an AT/RT-TYR tumor is shown in Fig. 1a). Subsequently, we quantified the percentage of ciliated cells for each of the samples and found that AT/RT-TYR tumors demonstrated the highest percentage of ciliated cells (range 12–22%), while AT/RT-MYC and AT/RT-SHH tumors showed a variable degree (range 4–29%) and the lowest proportion (range 2–6%) of cells with a primary cilium, respectively (Fig. 1b).

As we detected primary cilia to a variable degree across all subgroups, indicating that this cellular subpopulation might be generally important, we next aimed to determine the functional relevance of primary ciliogenesis in AT/RT biology. We selected suitable cell line models to investigate the role of primary cilia in AT/RT cells based on the expression levels of *kinesin family member* *3A* (*KIF3A*), since KIF3A is one subunit of the heterotrimeric motor protein kinesin-2 and, thus, is crucially involved in the assembly of primary cilia [25]. We performed RNA sequencing to investigate the expression levels of *KIF3A* in nine AT/RT cell lines. Given the fact that BT-12 and CHLA-266 showed the highest expression of *KIF3A* in comparison to the other AT/RT cell lines (Supplementary Fig. 1a), we chose these two cell lines for further investigation. Immunofluorescence with antibodies against pericentrin and acetylated tubulin detected primary cilia in BT-12 and CHLA-266 cells (Fig. 1c, d) and revealed that *KIF3A* expression seems to be correlated with cilia presence also in the other AT/RT cell lines examined (Supplementary Fig. 1a, b). Quantification of the percentage of ciliated cells revealed approximately 10% ciliated cells in each cell line (Fig. 1e).

In summary, these findings highlight that primary ciliogenesis is generally observed in AT/RTs with a subgroup-specific enrichment in the proportion of ciliated cells.

### Disruption of primary ciliogenesis decreases the oncogenic potential of AT/RT cells in vitro

**Fig. 2:**
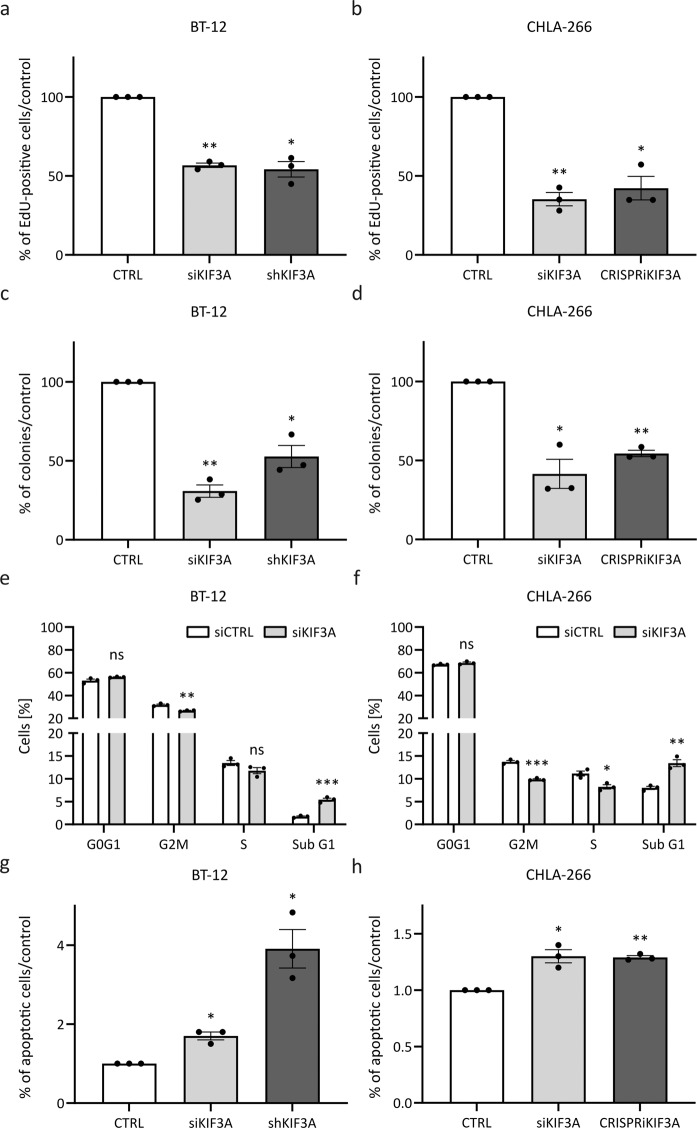
Knockdown of *kinesin family member 3A* (*KIF3A*) decreases the oncogenic potential of the AT/RT cell lines BT‐12 and CHLA‐266. Transient knockdown of *KIF3A* was achieved using siPOOLs. Stable knockdown of *KIF3A* was achieved using shRNA‐based (BT‐12) or CRISPRi‐based (CHLA‐266) technology. **a**, **b** Transiently or stably transfected BT‐12 (**a**) and CHLA‐266 (**b**) cells were incubated with 10 μM EdU (modified thymidine analogue) for 6 h. Cells were fixed and EdU was detected using Alexa Fluor™ 488 azide. Cells were counterstained with 4´,6‐diamidino‐2 phenylindole (DAPI). The percentage of proliferating cells was calculated based on the number of EdU‐labeled cells in relation to the number of DAPI‐labeled cells. **c**, **d** Quantification of colonies formed by transiently or stably transfected BT‐12 (**c**) and CHLA‐266 (**d**) cells following plating of 100 (BT‐12) or 500 (CHLA‐266) cells per 10 cm dish and 17 (BT‐12) or 24 (CHLA-266) days of incubation. **e**, **f** Cell cycle profiles of transiently transfected BT‐12 (**e**) and CHLA‐266 (**f**) cells. Cells were fixed and stained with propidium iodide (PI). DNA content was analyzed using flow cytometry. **g**, **h** To assess cells undergoing apoptosis, cells were stained with annexin V and PI and analyzed using flow cytometry. Shown are bar graphs representing the fold change in apoptotic cells following transient or stable *KIF3A* knockdown in the AT/RT cell lines BT-12 (**g**) and CHLA‐266 (**h**). Values shown represent mean ± SEM of three biologically independent replicates. ns: not significant, *p < 0.05; **p < 0.01; ***p < 0.001 (*t*-test).

To functionally investigate the role of primary cilia in AT/RT, we performed both siRNA-mediated (transient) and shRNA/CRISPRi-mediated (stable) *KIF3A* knockdown in BT-12 and CHLA-266 cells. Quantitative real-time PCR revealed that the expression of *KIF3A* mRNA was reduced following both transient and stable *KIF3A* knockdown in both cell lines (Supplementary Fig. 2a). Additionally, Western blot analysis confirmed reduced expression of KIF3A protein (Supplementary Fig. 2b). Immunofluorescence corroborated that the formation of primary cilia was decreased following both transient and stable *KIF3A* knockdown in both cell lines (Supplementary Fig. 2c, d). We then investigated the effects of disruption of primary ciliogenesis in BT-12 and CHLA-266 cells and observed a reduction in proliferation following both transient and stable *KIF3A* knockdown (Fig. 2a, b). Additionally, clonogenicity was reduced (Fig. 2c, d). Furthermore, cell cycle analysis revealed a decrease in the G2M fraction of cells, concomitant with an increase in the sub G1 fraction of cells (Fig. 2e, f), suggesting an increase in apoptotic cells. In line, we observed an increase in apoptotic cells following transient or stable *KIF3A* knockdown using Annexin V and PI staining (Fig. 2g, h).

**Fig. 3:**
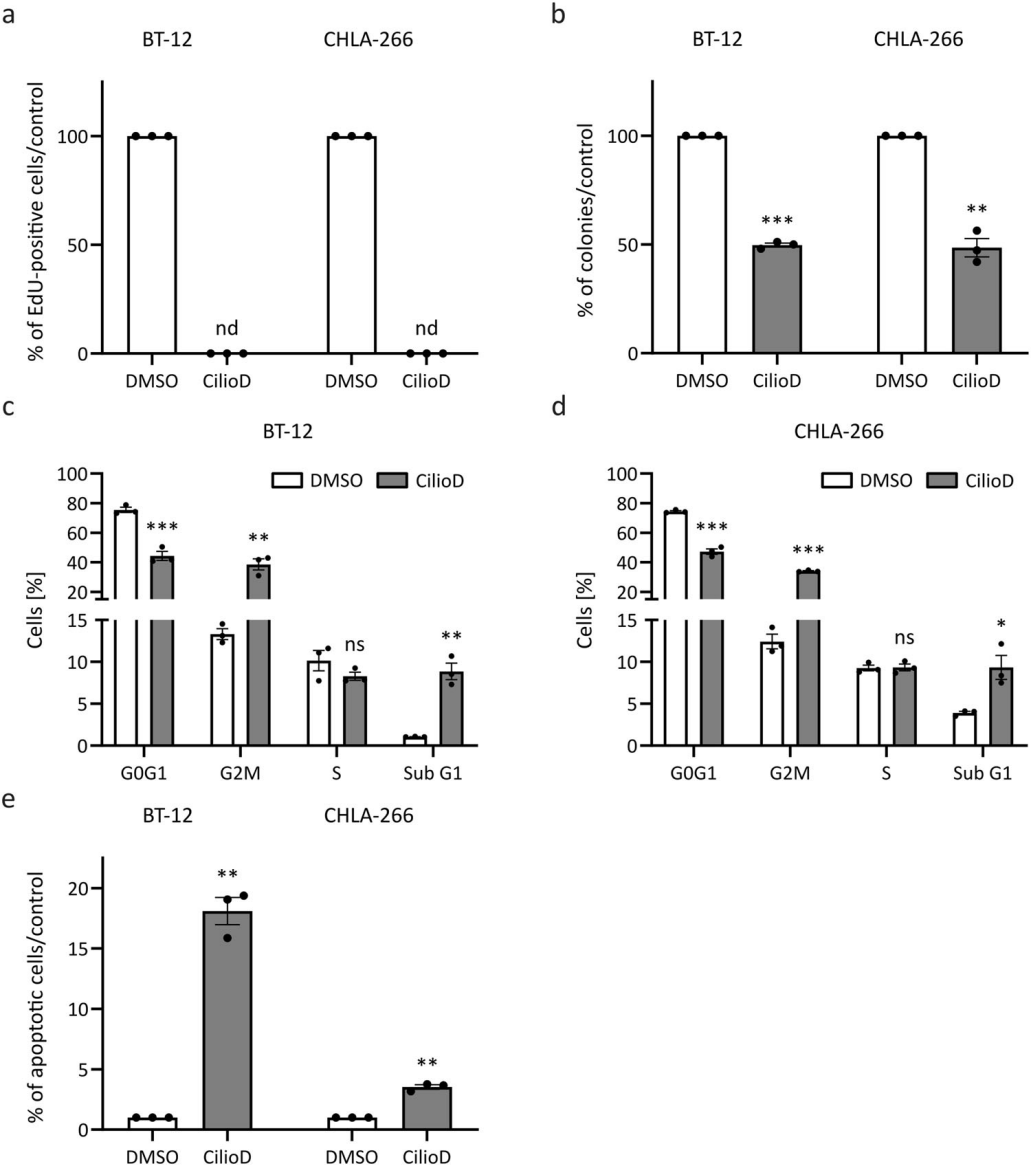
Ciliobrevin D (CilioD) treatment phenocopies *kinesin family member 3A* (*KIF3A*) knockdown in the AT/RT cell lines BT‐12 and CHLA‐266. BT‐12 and CHLA‐266 cells were seeded in complete medium. The next day, complete medium was replaced with medium lacking serum and the cells were treated with 30 µM CilioD or dimethylsulfoxide (DMSO) as a negative control. **a** CilioD treated BT‐12 and CHLA‐266 cells were incubated with 10 μM EdU for 6 h. Then, cells were fixed and EdU (modified thymidine analogue) was detected using Alexa Fluor™ 488 azide. Cells were counterstained with 4´,6‐diamidino‐2 phenylindole (DAPI). The percentage of proliferating cells was calculated based on the number of EdU‐labeled cells in relation to the number of DAPI‐labeled cells. **b** Quantification of colonies formed by CilioD treated BT‐12 and CHLA‐266 cells following plating of 100 (BT‐12) or 500 (CHLA‐266) cells per 10 cm dish and 17 (BT‐12) or 24 (CHLA‐266) days of incubation. **c**, **d** Cell cycle profiles of CilioD treated BT‐12 (**c**) and CHLA‐266 (**d**) cells. Cells were fixed and stained with propidium iodide (PI). DNA content was analyzed using flow cytometry. **e** To assess cells undergoing apoptosis, cells were stained with Annexin V and PI, and analyzed using flow cytometry. Shown are bar graphs representing the fold change in apoptotic cells following treatment with 30 μM of CilioD for 24 h or DMSO as a negative control in the AT/RT cell lines BT‐12 and CHLA‐266. Values shown represent mean ± SEM of three biologically independent replicates. nd: not detected, ns: not significant, *p < 0.05; **p < 0.01; ***p < 0.001 (*t*-test).

To functionally validate the anti-tumor effect upon genetic disruption of primary ciliogenesis, we employed a pharmacological approach using Ciliobrevin D (CilioD). CilioD is a cell-permeable, reversible and specific blocker of AAA+ ATPase motor cytoplasmic dynein that has been shown to perturb protein trafficking within the primary cilium and to block SHH signaling [26]. We observed complete loss of ciliated cells following CilioD treatment in both AT/RT cell lines (Supplementary Fig. 3e, f). Consistent with this finding, we found that the mRNA expression levels of *glioma-associated oncogene homolog 1* (*GLI1*), *patched 1* (*PTCH1*) and *smoothened* (*SMO*), three key SHH signature genes whose respective gene products are localized to the primary cilium, were reduced (Supplementary Fig. 3g, h). Additionally, we observed that CilioD treatment completely blocked proliferation (Fig. 3a) and decreased clonogenicity (Fig. 3b). Furthermore, cell cycle analysis revealed a decrease in the G0G1 fraction of cells, concomitant with an increase in the G2M and sub G1 fraction of cells (Fig. 3c, d) that also went along with an increase in apoptotic cells (Fig. 3e) following CilioD treatment.

Overall, these results demonstrate that primary ciliogenesis promotes tumorigenicity of AT/RT in vitro.

### Proteogenomic profiling reveals induction of interferon signaling following disruption of primary ciliogenesis in AT/RT cells

**Fig. 4:**
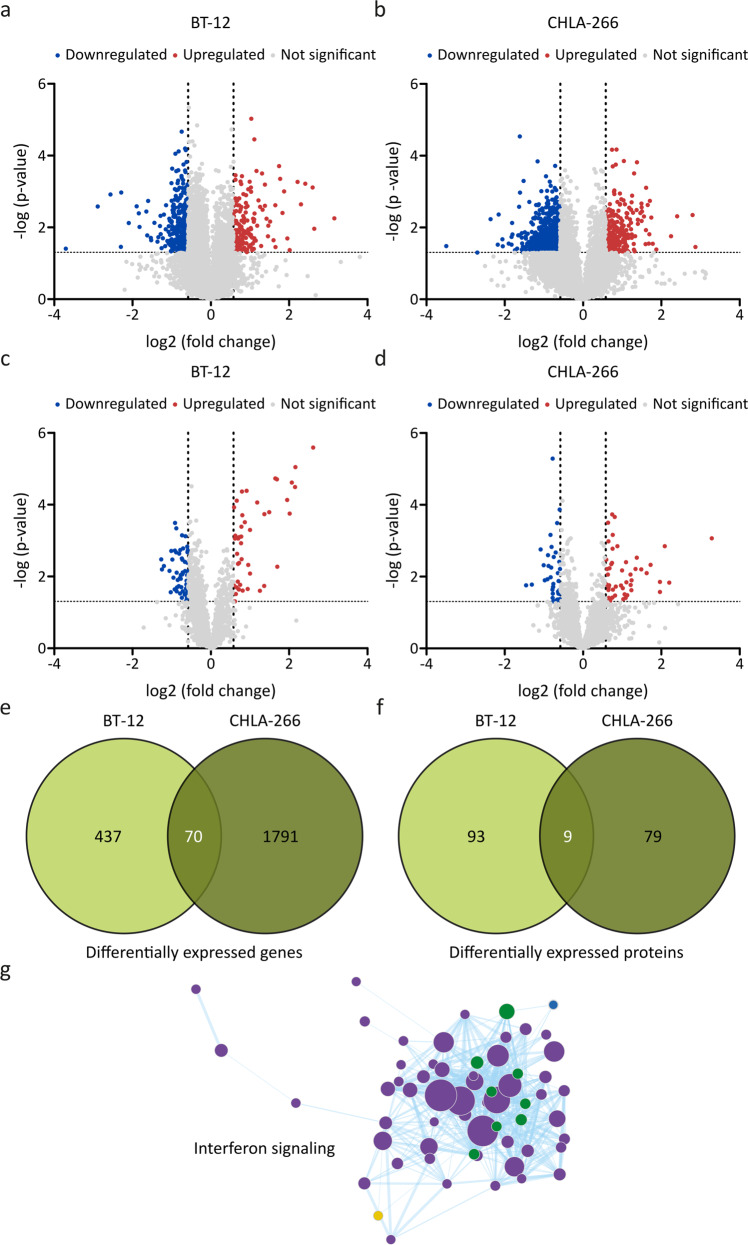
Proteogenomic profiling reveals induction of interferon signaling following *kinesin family member 3A* (*KIF3A*) knockdown in the AT/RT cell lines BT‐12 and CHLA‐266. **a**–**d** Volcano plots showing downregulated (blue dots), upregulated (red dots) and not significant (grey dots) transcripts (**a**, **b**) and proteins (**c**, **d**) following *KIF3A* knockdown in BT‐12 (**a**, **c**) and CHLA‐266 (**b**, **d**) cells, using a minimal fold change of ± 1.5 and a significance level of *p* < 0.05 as a cut‐off. Statistical analysis was performed using the non‐parametric Kruskal Wallis test. **e**, **f** Venn diagrams showing the overlap of differentially regulated genes (**e**) and proteins (**f**) between BT‐12 and CHLA‐266 cells. **g** Gene set enrichment analysis (GSEA) was performed for merged RNA sequencing and proteomics data. Green circles: Overlap between mass spectrometry (MS) and RNA sequencing (RS) upregulated in *KIF3A* knockdown cells. Purple circles: Upregulated in MS *KIF3A* knockdown cells. Blue circles: Upregulated in RS *KIF3A* knockdown cells. Yellow circle: Upregulated in MS control cells.

To elucidate the biological mechanism that could be responsible for the anti-tumor effects following disruption of primary ciliogenesis, we conducted comprehensive analysis of the transcriptome and the proteome following transient *KIF3A* knockdown in BT-12 and CHLA-266 cells. Using a minimal fold change of ± 1.5 and a significance level of *p* < 0.05 as cut-off criteria, a total of 507 and 1861 differentially expressed genes, as well as 102 and 88 differentially expressed proteins were identified in BT-12 and CHLA-266 cells, respectively (Fig. 4a–f). Amongst these, a total of 70 genes and 9 proteins were differentially expressed following *KIF3A* knockdown in both cell lines (Fig. 4e, f; Supplementary Tables 2, 3).

To elucidate canonical pathways and upstream regulators controlled by *KIF3A* knockdown, we performed ingenuity pathway analysis (IPA) on the 70 genes and 9 proteins that were differentially expressed in BT-12 and CHLA-266 cells. IPA analysis of the transcriptomic data identified three canonical pathways as being significantly dysregulated, namely thiamin salvage III, interferon signaling and the inflammasome pathway (Supplementary Table 4). Amongst the significantly dysregulated upstream regulators, almost all (9 of 11, 82%) were functionally linked to lipid biosynthesis and metabolism, while two of the 11 upstream regulators (18%) were functionally linked to interferon signaling (Supplementary Table 5). We identified 37 canonical pathways as being significantly dysregulated using the proteomic data. Specifically, 32 out of the 37 canonical pathways (86%) were functionally linked to interferon signaling. Notably, interferon signaling was the most affected canonical pathway in this dataset (Supplementary Table 6). In addition, most upstream regulators (24 of 31, 77%) were functionally linked to interferon signaling as well (Supplementary Table 7).

**Fig. 5:**
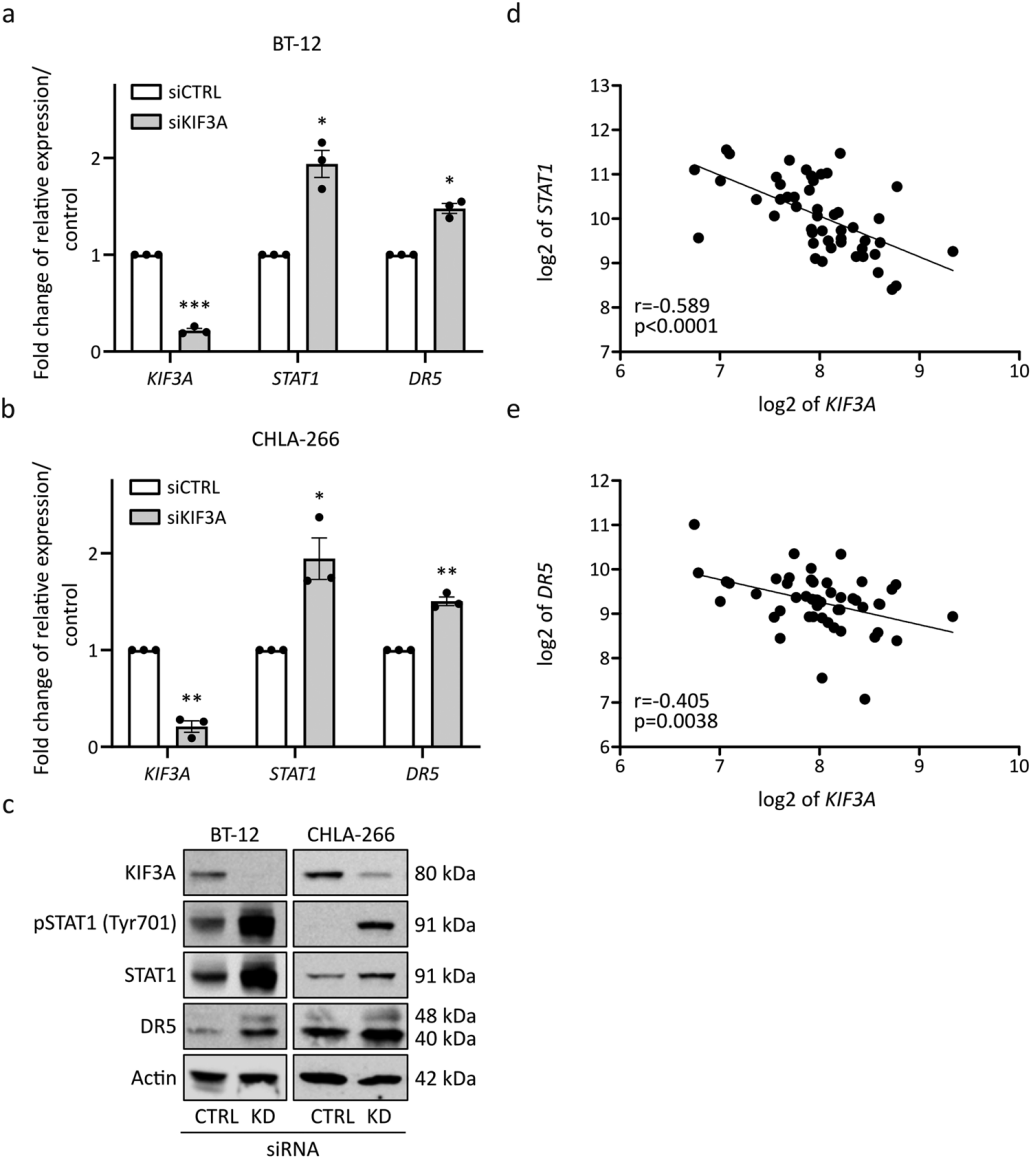
Validation of upregulated signal transducer and activator of transcription 1 (STAT1) and death receptor 5 (DR5) signaling following *kinesin family member 3A* (*KIF3A*) knockdown in the AT/RT cell lines BT‐12 and CHLA‐266. **a**, **b** siRNA‐mediated knockdown of *KIF3A* in BT‐12 (**a**) and CHLA‐266 (**b**) cells was achieved using siPOOLs. mRNA expression was normalized to the housekeeping genes *glyceraldehyde 3‐phosphate dehydrogenase* (*GAPDH*) and *phosphoglycerate kinase 1* (*PGK1*) and calculated relative to siCTRL. Values shown represent mean ± SEM of three biologically independent replicates. *p < 0.05; **p < 0.01; ***p < 0.001 (*t*‐test). **c** Representative Western blot images for KIF3A, pSTAT1 (Tyr701), STAT1, DR5 and actin as a loading control following siRNA‐mediated *KIF3A* knockdown. **d**, **e** Gene expression data are derived from the Tumor ATRT ‐ Kool ‐ 49 ‐ MAS5.0 ‐ u133p2 data set available at the R2: Genomics analysis and visualization platform (http://r2.amc.nl).

Considering both the transcriptomic and the proteomic data, the only overlap observed between both data sets across both cell lines was a significant induction of interferon signaling, as detected by gene set enrichment analysis (GSEA, Fig. 4g). Several key signaling cascades are essential for the induction of interferon responses [27]. In this study, we specifically observed a significant upregulation of STAT1 and DR5 signaling following *KIF3A* knockdown in BT-12 and CHLA-266 cells. Validation by quantitative real-time PCR revealed an increased expression not only of *STAT1* and *DR5* mRNA levels (Fig. 5a, b), but also of several STAT1-induced transcripts (*OAS1*, *OAS2*, *OAS3*, *MX1*, *IFIT1*, *IFIT3*, *IFI35*, and *ISG15*, Supplementary Fig. 4). Additionally, Western blot analysis corroborated increased STAT1 protein expression and STAT1 phosphorylation on residue 701. Moreover, we observed an increased DR5 expression (Fig. 5c). Analysis of AT/RT patient data through the web-based genomics analysis and visualization platform R2 (http://r2.amc.nl) confirmed an anti-correlated expression between *STAT1* and *DR5* transcripts (Fig. 5d, e), as well as all other above-mentioned STAT1-induced genes when compared to the *KIF3A* transcript levels (Supplementary Fig. 5).

Altogether, these findings highlight a role for primary cilia in suppressing STAT1 and DR5 signaling and, thus, suppressing apoptosis.

### Primary ciliogenesis contributes to AT/RT biology in vivo

**Fig. 6:**
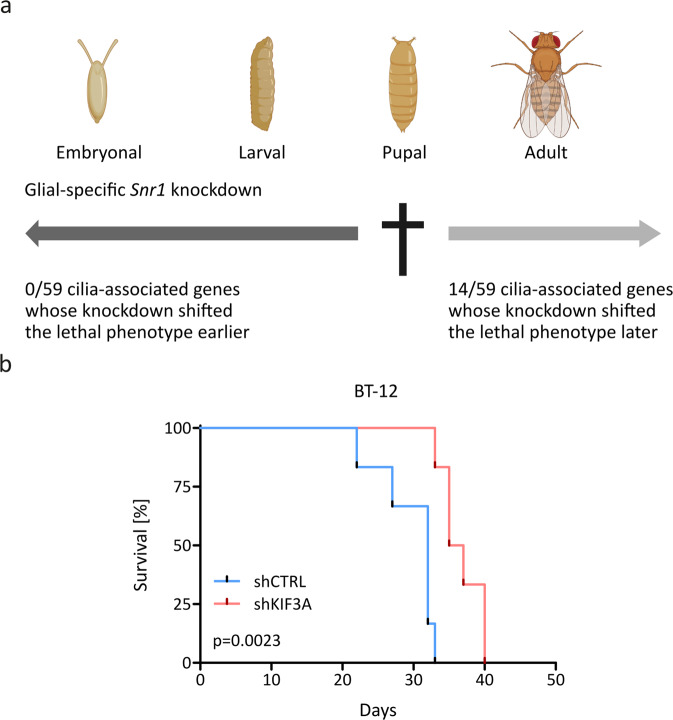
Primary ciliogenesis contributes to AT/RT biology in vivo. **a** Crossing of *Snf5‐related 1* (*Snr1*) knockdown flies with strains expressing specific RNA interference (RNAi) shifted the pupal lethal phenotype associated with glial‐specific *Snr1* knockdown to later stages of development in 14 out of 59 screened candidate genes. The complete list of the 59 genes is shown in Supplementary Table 8. Created with BioRender.com. **b** Kaplan‐Meier survival curve of NOD scid gamma (NSG) mice orthotopically injected with control BT‐12 cells (*n* = 6, shCTRL, blue) or *KIF3A* knockdown BT‐12 cells (*n* = 6, shKIF3A, red). Statistical analysis was performed using the Gehan‐Breslow‐Wilcoxon test.

Given the anti-tumor effects of *KIF3A* knockdown in vitro, we also investigated the role of primary cilia in AT/RT biology in vivo. To this aim, we used a *Drosophila* model of glial-specific *Snf5-related 1* (*Snr1*, *Drosophila* orthologue of *SMARCB1*) deficiency, which leads to death at the pupal stage of development [28] and, therefore, allows for high-throughput screens of genes functionally involved in the lethal phenotype of *Snr1* deficiency. In order to investigate the role of primary ciliogenesis in this *Drosophila* model, we crossed *Snr1* knockdown flies with strains expressing specific RNAi species for 59 genes associated with ciliogenesis (the complete list of the 59 genes is shown in Supplementary Table 8). We then conducted a screen for a shift in the lethal phenotype. Notably, additional knockdown of 14 out of the 59 examined cilia-associated genes (24%) resulted in a substantial shift of the lethal phenotype with more than 20% of flies reaching adulthood (Fig. 6a). We also evaluated an orthotopic xenograft mouse model of AT/RT using BT-12 shCTRL and BT-12 shKIF3A cells injected in the right hemisphere of the frontal cortex of NSG mice. Knockdown of *KIF3A* resulted in a significantly prolonged median survival of mice compared to the control (Fig. 6b).

Taken together, these results provide compelling evidence that primary ciliogenesis promotes tumorigenicity of AT/RT in vivo.

## Discussion

The knowledge of the roles of primary cilia in cancer pathogenesis is increasingly growing. Overall, the frequency of primary cilia is lower in tumor tissues compared to their respective control tissues [29]. However, the functional implication of primary ciliogenesis is highly divergent, since primary cilia have been reported to have dual and opposing roles in different tumor types and within tumor subtypes. Specifically, they can act as both tumor suppressors and oncogenic drivers [17]. Using an immunofluorescence approach, we were able to detect primary cilia in all investigated AT/RT primary tumor samples and cell lines. However, we observed a subgroup-specific difference in the percentage of ciliated cells, with AT/RT-TYR tumors demonstrating the highest percentage of ciliated cells. Since SHH signaling is the best-studied pathway linked to primary cilia [30, 31], it was rather unexpected that AT/RT-SHH tumors showed the lowest proportion of cells with a primary cilium. However, this might be explained by the hypothesis that AT/RT-SHH tumors rely on downstream activation of SHH signaling and, therefore, are independent of primary cilia for constitutive active SHH signaling as previously shown for the dual and opposing role for primary cilia in SHH-dependent medulloblastoma and basal cell carcinoma [22, 32], depending on the initiating oncogenic event. The finding that the AT/RT-TYR subgroup demonstrates the highest percentage of ciliated cells is in line with a recent large-scale multicenter study reporting that many genes involved in ciliogenesis are highly expressed in this subgroup [11].

To investigate the functional roles of primary cilia in AT/RT, we disrupted primary ciliogenesis in vitro in two AT/RT cell line models using two different but complementary approaches. Previous studies have shown that disruption of primary ciliogenesis may be achieved by genetic ablation of *KIF3A* [22, 32–34], which we were able to confirm for our AT/RT cell line models. Knockdown of *KIF3A* resulted in a significant reduction of tumor-promoting properties. This is in line with previous studies reporting on tumor growth arrest upon *KIF3A* ablation in medulloblastoma and glioblastoma cells [33, 34]. We further validated these findings by disrupting primary ciliogenesis pharmacologically using CilioD, a specific small‐molecule antagonists of cytoplasmic dynein [26]. Overall, we observed that CilioD treatment widely phenocopied the results from the *KIF3A* knockdown experiments, suggesting that primary cilia contribute to the aggressive phenotype of AT/RT cells in vitro.

Primary cilia play a critical role throughout central nervous system development, from early patterning, through the proliferation and differentiation of neural stem and progenitor cells, to the maturation of neurons [35]. Thus, potential side effects of drugs inhibiting primary cilia function remain to be determined. The relevance of primary ciliogenesis in AT/RT biology was also confirmed in vivo using a *Drosophila* model of *SMARCB1* deficiency and an orthotopic xenograft mouse model of AT/RT, emphasizing its cross-species function. Conducting a high-throughput screen of genes functionally involved in the lethal phenotype of *Snr1* (*Drosophila* orthologue of *SMARCB1*) deficiency, we found that the proportion of cilia-associated genes causing a positive shift of the phenotype was significantly higher compared to the proportion previously reported for a large set of genes with known nervous system expression [28] (Supplementary Fig. 6), suggesting that cilia-associated genes have an important role in the detrimental effects of *Snr1* knockdown in *Drosophila*. We validated this finding in an orthotopic xenograft mouse model of AT/RT by showing that *KIF3A* knockdown significantly extended the survival of recipient mice compared to corresponding isogenic controls.

Elucidating the biological mechanism that could be responsible for the anti-tumoral effect following disruption of primary ciliogenesis, we performed proteogenomic profiling following *KIF3A* knockdown in two AT/RT cell line models. We and others could already demonstrate the value of integrated proteogenomic approaches, showing that transcriptomic profiles combined with proteomic profiles provide profound insights into active oncogenic gene expression regulation in pediatric brain tumors and other cancers [36–40]. In the present study, we uncovered a significant induction of only one canonical pathway, namely interferon signaling. Specifically, we observed an increase in STAT1 signaling, with concomitant upregulation of DR5, suggesting that primary cilia suppress apoptosis induction in AT/RT. To date, a direct link between primary cilia and STAT1 or DR5 signaling in cancer has not been reported. In several types of cancers, including colorectal carcinoma [41], pancreatic cancer [42], soft tissue sarcoma [43] and metastatic melanoma [44], STAT1 expression has been correlated with cell cycle inhibition and sensitization to apoptotic stimuli. STAT1 may induce different forms of cell death by transcriptionally regulating the expression of cell cycle regulators [45], pro-apoptotic proteins [46] and death receptors and their ligands [47]. Recently, Chun et al. showed an enrichment of transcription factor binding sites in the enhancers of genes involved in apoptosis and immune regulation in bulk tumor samples of AT/RT-MYC, such as *GMEMB1/2*, *RAD21*, *IRF5/8/9*, and *STAT1* [48]. The individual proportion of ciliated cells was not determined in this or previous studies [10, 11] and CD8+ cytotoxic T cells were remarkably increased in AT/RT-MYC [48]. Single cell RNA sequencing analyses will be needed to determine whether this enrichment may be explained by differences in primary ciliogenesis or are potentially due to distinct immune cell infiltration patterns.

In summary, we show that primary cilia contribute to the malignant phenotype of AT/RT both in vitro and in vivo. Specifically, we indicate a role for primary cilia in suppressing apoptosis, potentially resulting in resistance to the current treatment approaches observed in a significant proportion of patients. Hence, targeting primary ciliogenesis or its downstream effects may provide novel therapeutic avenues for this highly aggressive brain tumor in infants.

## Methods

### Patient samples

AT/RT samples were retrieved from the archives of the Institute of Neuropathology at the University Hospital Münster, Germany. For molecular characterization of the samples, DNA methylation array profiling was performed at the German Cancer Research Center in Heidelberg, Germany, using the Infinium HumanMethylation450 BeadChip from Illumina, San Diego, CA, USA, as described previously [11].

### Cell cultivation

AT/RT cell lines BT-12 and CHLA-266 were kindly provided by the Children’s Oncology Group (COG) Cell Culture and Xenograft Repository (Lubbock, TX, USA) and maintained as monolayer cultures in complete medium, consisting of Iscove’s modified dulbecco’s medium (IMDM, Gibco, Darmstadt, Germany, #12440053) supplemented with 20% fetal bovine serum (FBS, Sigma‐Aldrich, Taufkirchen, Germany #F9665) and 1x Insulin‐Transferrin‐Selenium (ITS, Gibco, #41400045). HEK293T cells were obtained from the American Type Culture Collection (ATCC, Manassas, VA, USA) and were maintained as monolayer cultures in complete medium, consisting of Dulbecco’s modified eagle’s Medium (DMEM, Gibco, #31966021) supplemented with 10% FBS. Cells were incubated at 37 °C in a 5% CO_2_ atmosphere. All cell lines were authenticated by short tandem repeat (STR) profiling and tested for mycoplasma contamination.

### Immunofluorescence

For the detection of primary cilia, AT/RT tissues and cell lines were stained with a polyclonal rabbit antibody against pericentrin (Abcam, Cambridge, UK, #ab4448, 1:500) or a mouse monoclonal antibody against acetylated tubulin (6-11B-1, Sigma‐Aldrich, #T6793, 1:500) as primary antibodies, detecting the basal body and the axoneme of the primary cilium, respectively. Secondary antibodies were species-specific and conjugated with fluorescence tags (Thermo Fisher Scientific, Bremen, Germany, Chicken anti‐Rabbit IgG, Alexa Fluor 488, #A‐21441, and Goat anti‐Mouse IgG2b, Alexa Fluor 594, #A‐21145, each 1:2500). AT/RT cell lines grown on coverslips were fixed and permeabilized with 4% formaldehyde solution (Thermo Fisher Scientific, #28908) supplemented with 0.1% Triton X-100 (Roth, Karlsruhe, Germany, #6683.1) in PBS-T for 20 min at room temperature (RT). In order to block unspecific binding sites, cells were incubated with 1% BSA (Roth, #8076.4) and 3% goat serum (Sigma‐Aldrich, #G9023) in PBS-T for 1 h at RT. Subsequently, cells were incubated with primary antibodies diluted in blocking solution overnight at 4 °C. The next day, cells were incubated with secondary antibodies diluted in blocking solution for 45 min at RT and counterstained with 4´,6‐diamidino‐2‐phenylindole (DAPI, Thermo Fisher Scientific, #62248, 1:1000) diluted in PBS for 3 min at RT. Finally, coverslips were mounted on glass slides using Vectashield Mounting Medium for Fluorescence (Vector Laboratories, Eching, Germany, #H‐1000) and Eukitt Quick-Hardening Mounting Medium (Fluka, Schwerte, Germany, #3989). Paraffin AT/RT tissue sections were deparaffined by two-time incubation in 100% xylene (Roth, #9713.3) for 5 min at RT followed by the incubation in an alcohol dilution series at RT (three-time incubation in 100% EtOH (VWR Chemicals, Fontenay‐sous‐Bois, France, #20.821.330) for 3 min, incubation in 95% EtOH for 2 min was conducted twice and followed by incubation in 70% EtOH for 1 min). Subsequently, tissue sections were boiled in citrate solution (100 mM Citrate (pH 6.0) dissolved in ddH_2_O) for antigen retrieval for 30 min. Blocking of unspecific binding sites and staining for primary cilia was performed as described above. Before mounting, tissue sections were incubated in 1% Sudan Black B (Sigma‐Aldrich, #199664) in 70% EtOH for 10 min at RT and washed with 70% EtOH afterwards in order to diminish autofluorescence. Stained coverslips and tissue sections were analyzed using the Axio Observer.Z1 with a 63x oil objective and the ApoTome.2 from Zeiss.

### Quantification of ciliated cells

For the quantification of ciliated cells, at least three randomly selected microscopic fields per coverslip/tissue section were examined. All images were captured as z-stacks of at least 20 images. The percentage of ciliated cells was calculated by counting the number of DAPI-labeled cells and the number of primary cilia and calculating the ratio afterwards.

### RNA interference using small interfering RNA (siRNA)

Transient knockdown of *KIF3A* was achieved using small interfering RNA (siRNA) pools (siPOOLs) [49]. KIF3A siPOOLs (siKIF3A, #11127) and negative control siPOOLs (siCTRL) were obtained from siTOOLs Biotech, Martinsried, Germany. BT-12 (2.5 × 10^5^ cells/well) and CHLA-266 (5 × 10^5^ cells/well) cells were seeded on 6-well plates. The next day, transfection of siPOOLs was performed using Lipofectamin® RNAiMAX Reagent (Invitrogen, Darmstadt, Germany, #13778150) according to the manufacturer’s instructions.

### Generation of stable cell lines

Stable knockdown of *KIF3A* was achieved using shRNA-based technology in BT-12 cells and CRISPRi-based technology in CHLA-266 cells.

### RNA interference using short hairpin RNA (shRNA)

The lentiviral vector backbone pLK0.1 - TRC was obtained from AddGene, Watertown, MA, USA (#10878). A set of shRNA oligos was obtained from IDT Integrated DNA Technologies, Coralville, IA, USA (see Supplementary Table 9).

### CRISPR interference (CRISPRi)

The lentiviral vector backbone pLV hU6-sgRNA hUbc-dCas9-KRAB-T2a-Puro was a kind gift from Charles Gersbach (Duke University, Durham, NC). Single guide RNAs (sgRNAs) were designed on the basis of Guide Design Resources (http://crispr.mit.edu/). A set of sgRNA oligos was obtained from IDT Integrated DNA Technologies (see Supplementary Table 9).

### Virus production

For the production of lentiviral particles, HEK293T cells were transfected with lentiviral vector, as well as helper and envelope plasmids using polyethylenimine (PEI, Merck, Darmstadt, Germany, #408727). The medium was replaced with fresh medium 24 h after the transfection. The lentiviral-containing medium was collected and filtered through a 0.45 µm filter 24 h after the medium change. Target cells were directly infected with the virus solution using 2 µg/ml polybrene (Merck, Darmstadt, Germany, #TR-1003-G). Stably transduced cells were selected using 0.5 µg/ml puromycin (InvivoGen, San Diego, CA, USA, #ant‐pr‐1).

### Ciliobrevin D (CilioD) treatment

CilioD is only effective upon serum deprivation in BT-12 and CHLA-266 cells (Supplementary Fig. 3a, b), since the percentage of ciliated cells is significantly increased under these conditions (Supplementary Fig. 3c, d). Therefore, all experiments that include CilioD treatment were performed under serum starvation conditions. For this, BT-12 and CHLA-266 cells were seeded in complete medium. The next day, complete medium was replaced with medium lacking serum. Then, 30 µM of CilioD (Calbiochem, Darmstadt, Germany, #250401) dissolved in dimethylsulfoxide (DMSO, AppliChem, Darmstadt, Germany, #A3672) or DMSO as negative control were added. After 72 h, cell viability was determined using the CellTiter-Glo Luminescent Cell Viability Assay (Promega, Madison, WI, USA, #G7572) according to the manufacturer’s instructions. The CellTiter-Glo Reagent was diluted with PBS (1:2, v/v).

### Proliferation

Either stably transduced or parental BT-12 (1 × 10^4^ cells/well) and CHLA-266 (2 × 10^4^ cells/well) cells were seeded on 8-well glass slides (Thermo Fisher Scientific, #154534). The next day, parental cells were either transfected using siPOOLs as described above or treated with 30 µM of CilioD dissolved in DMSO or DMSO as negative control. After 72 h or 24 h, cell proliferation was determined using the Click-iT EdU Alexa Fluor 488 Imaging Kit (Invitrogen, #C10337) according to the manufacturer’s instructions and analyzed using the Axio Observer.Z1 with a 40x oil objective and the ApoTome.2 from Zeiss.

### Clonogenicity

In case of siRNA-mediated transfection, parental BT-12 or CHLA-266 cells were transfected with siPOOLs as described above. After 48 h, transfected BT-12 (100 cells/well) and CHLA-266 (500 cells/well) cells were harvested and seeded on 10 cm dishes. In case of lentiviral transduction, stably transduced BT-12 (100 cells/well) and CHLA-266 (500 cells/well) cells were seeded on 10 cm dishes. In case of CilioD treatment, parental BT-12 (100 cells/well) and CHLA-266 (500 cells/well) cells were seeded on 10 cm dishes. The next day, cells were treated with 30 µM of CilioD dissolved in DMSO or DMSO as negative control. After 24 h, medium containing CilioD or DMSO was replaced with fresh medium. After 17 days (BT-12) or 24 days (CHLA-266), cells were fixed with 10% formaldehyde (ScyTek Laboratories, Hamburg, Germany, #FRN999) for 30 min at RT and stained with 0.1% crystal violet (Fluka, Schwerte, Germany, #32675) for 1 h at RT.

### Cell cycle and apoptosis

Either stably transduced or parental BT-12 (1.25 × 10^5^ cells/well) and CHLA-266 (2.5 × 10^5^ cells/well) cells were seeded on 12-well plates. The next day, parental cells were either transfected using siPOOLs as described above or treated with 30 µM of CilioD dissolved in DMSO or DMSO as negative control. After 72 or 24 h, cells were harvested and propidium iodide (PI, Sigma‐Aldrich, #P4864) or Annexin V staining was performed. For PI staining, cells were fixed with ice cold EtOH (70%) for at least 30 min at 4 °C. Then, cell pellets were resuspended in 50 µl RNase A (0.1 mg/ml, AppliChem, #A3832), followed by the addition of 150 µl PI (50 µg/ml). Cells were stained overnight at 4 °C. For Annexin V staining, cells were stained with FITC Annexin V (BD Biosciences, San Jose, CA, USA, #556419 (FITC Annexin V), #556454 (Annexin V Binding Buffer)) and PI according to the manufacturer’s instructions. For both assays, cells were analyzed using the CytoFLEX from Beckman Coulter.

### RNA extraction, cDNA synthesis and quantitative real-time PCR

RNA was extracted using TRIzol® Reagent (Thermo Fisher Scientific, #15596018) and cDNA was synthesized from 0.5 µg of total RNA using M-MLV reverse transcriptase (Promega, #M3681) according to the manufacturer’s instructions. Gene expression was determined by quantitative real-time PCR using the CFX384^TM^ Real-Time System from Bio-Rad. *Glyceraldehyde 3‐phosphate dehydrogenase* (*GAPDH*) and *phosphoglycerate kinase 1* (*PGK1*) served as housekeeping genes for normalization. Relative quantification of PCR products was conducted using the ΔΔCT method. Samples were quantified in triplets. Primers for GoTaq® real‐time PCR (see Supplementary Table 10) and for TaqMan® real‐time PCR (see Supplementary Table 11) were obtained from IDT Integrated DNA Technologies.

### RNA sequencing

#### Sample preparation

Cells were transfected with siPOOLs as described above for 72 h. RNA was extracted as described above and was processed using the TruSeq RNA Sample Preparation v2 Kit (low-throughput protocol; Illumina, San Diego, USA) to prepare the barcoded libraries from 0.5 µg total RNA. Libraries were validated and quantified using DNA 1000 and high-sensitivity chips on a Bioanalyzer (Agilent, Böblingen, Germany); 7.5 pM denatured libraries were used as input into cBot (Illumina), followed by deep sequencing using HiSeq 2500 (Illumina) for 101 cycles, with an additional seven cycles for index reading.

#### Data analysis

Fastq files were imported into Partek Flow (Partek Incorporated, Missouri, USA). Quality analysis and quality control were performed on all reads to assess read quality and to determine the amount of trimming required (both ends: 13 bases 5´and 1 base 3´). Two samples were excluded from the analysis since they did not pass the quality control. Trimmed reads were aligned against the hg38 genome using the STAR v2.4.1d aligner. Unaligned reads were further processed using Bowtie 2 v2.2.5 aligner. Aligned reads were combined before quantifying the expression against the ENSEMBL (release 84) database by the Partek Expectation-Maximization algorithm. Finally, statistical gene set analysis was performed using the non-parametric Kruskal Wallis test to determine differential expression at the gene level. Partek flow default settings were used in all analyses.

#### Pathway analysis

Ingenuity pathway analysis (IPA, Qiagen, Hilden, Germany) was conducted using genes with significant differential expression (fold change ± 1.5 and *p* ≤ 0.05). The significance cut-off for IPA was set to *p* < 0.05 for identification of canonical pathways and upstream regulators. Heatmap visualization and unsupervised hierarchical clustering were performed after normalizing mean expression to 0 with a standard deviation of 1 and using Pearson’s dissimilarity algorithm and average linkage in Partek Genomics Suite (Partek Incorporated). Raw data are available online at NCBI GenBank (accession number: GSE179668).

### Cell lysis, protein extraction and Western blot analysis

Cells were lysed and protein was extracted using RIPA Lysis Buffer (Sigma‐Aldrich, #20‐188) supplemented with protease (Roche, Basel, Switzerland, #4693132001) and phosphatase (Roche, #4906837001) inhibitor cocktail. Protein was quantified with the Bradford method using the Protein Assay Dye Reagent Concentrate (Bio‐Rad, Hercules, CA, USA, #500‐0006) [50]. Samples were separated by SDS polyacrylamide gel electrophoresis (SDS-PAGE) using Novex WedgeWell 4–12% Tris-Glycine Gels (Invitrogen, #XP04122BOX) and transferred to Amersham Protran 0.45 µm nitrocellulose membranes (GE Healthcare, Chicago, IL, USA, #10600002) by wet blotting using the Mini Gel Tank and Blot Module from Thermo Fisher Scientific. In order to block unspecific binding sites, membranes were incubated with 5% BSA in TBS-T for 1 h at RT. Subsequently, membranes were incubated with primary antibodies (EMD Millipore, Burlington, VT, USA, Actin (C4), #MAB1501, 1:5000, Cell Signaling Technology, Cambridge, UK, DR5 (D4E9) XP® rabbit mAB, #8074, KIF3A (D763) rabbit mAB, #8507, Phospho-STAT1 (Tyr701) (58D6), #9167, STAT1 rabbit pAB, #9172, each 1:1000) diluted in blocking solution overnight at 4 °C. The next day, membranes were incubated with secondary antibodies (Cell Signaling Technology, anti-mouse IgG, HRP-linked, #7076, or anti-rabbit IgG, HRP-linked, #7074, each 1:5000) diluted in blocking solution for 1 h at RT. Proteins were visualized using the SuperSignal West Femto Maximum Sensitivity Substrate (Thermo Fisher Scientific, #C118A) and detected using the LAS-3000 Imaging System from Fujifilm.

### Proteomic profiling

#### Sample preparation

Cells were transfected with siPOOLs as described above for 72 h. Proteins were extracted as described [51]. Briefly, cells were homogenized in urea buffer with a TissueLyser (Qiagen, Hilden, Germany) and subsequent sonication. After centrifugation for 15 min at 14000 x g and 4 °C, supernatants were collected. Protein concentration was determined via Pierce 660 nm Protein Assay (Thermo Fisher Scientific) and 10 µg protein per sample were desalted through electrophoretic migration at 50 V for 10 min on a 4–12% Bis-Tris polyacrylamide gel. After silver staining, protein bands were cut out reduced, alkylated and digested with trypsin before peptide extraction via sonication. Generated peptide samples were diluted with 0.1% TFA at a ratio of 1:8.

#### LC-MS analysis

For mass spectrometric analysis, 15 µl peptide solution per sample was analyzed on a nano-high-performance liquid chromatography electrospray ionization mass spectrometer. The analytical system was composed of a RSLCnano U3000 HPLC coupled to an Orbitrap Elite or a QExactive plus mass spectrometer via a nano-electrospray ion source (Thermo Fischer Scientific). Injected peptides were concentrated and desalted at a flow rate of 6 µl/min on a trapping column (Acclaim PepMao C18, 2 cm × 100 µm x 3 µm particle size, 100 Å pore size, Thermo Fischer Scientific) with 0.1% TFA for 10 min. Subsequently, peptides were separated at a constant flowrate of 300 nl/min over a 120 min gradient on an analytical column (Acclaim PepMap RSLC C18, 25 cm × 75 µm x 2 µm particle size, 100 Å pore size, Thermo Fischer Scientific) at 60 °C. Separation was achieved through a gradient from 4 to 40% solvent B (solvent A: 0.1% (v/v) formic acid in water, solvent B: 0.1% (v/v) formic acid, 84% (v/v) acetonitrile in water). Afterwards, peptides were ionized at a voltage of 1400 V and introduced into the mass spectrometer operated in positive mode. On the Orbitrap Elite, MS scans were recorded in profile mode in a range from 350 to 1700 m/z at a resolution of 60000 while tandem mass spectra were recorded in the ion trap at normal scan rate. Tandem mass spectra were recorded with a data dependent Top20 method and 35% normalized collision energy. Dynamic exclusion was activated with a repeat count of 1 for 45 s and only charge states 2 + and 3 + were analyzed. MS scans on the QExactive plus were recorded in profile mode in a range from 350–2000 m/z at a resolution of 70000, while tandem mass spectra were recorded at a resolution of 17500. Tandem mass spectra were recorded with a data dependent Top10 method and 30% normalized collision energy. Dynamic exclusion was activated with a repeat count of 1 for 100 ms.

#### Computational mass spectrometric data analysis

Proteome Discoverer (version 2.3.0.523, Thermo Fisher Scientific) was applied for peptide/protein identification with Mascot and MS Amanda as search engines employing the UniProt database (human; including isoforms; date 2019-05-29). A false discovery rate of 1% (*p* ≤ 0.01) on peptide level was set as identification threshold. Proteins were quantified with Progenesis QI for Proteomics (Version 2.0, Nonlinear Dynamics, Waters Corporation, Newcastle upon Tyne, UK). The mass spectrometry proteomics data have been deposited to the ProteomeXchange Consortium via the PRIDE [52] partner repository with the dataset identifier PXD027211.

### *Drosophila melanogaster* experiments

#### Fly husbandry

All strains were raised on corn meal yeast agar and kept at 25 °C and 60% humidity. The following fly strains were used:

$$\frac{{repo - GAL4,UAS - mCD8GFP}}{{repo - GAL4,UAS - mCD8GFP}};\frac{{repo - GAL4,UAS - mCD8GFP}}{{TM6\;B}}$$

(provided by Prof. Dr. Christian Klämbt, Institute of Neurobiology, University Münster, Münster, Germany),

$$\frac{ + }{ + };\frac{{TM2}}{{tubulin - GAL80,TM6,\;Tb}}$$

(provided by Harvard *Drosophila* Stock Collection, Boston, USA),

$$\frac{{UAS - Snr1 - RNAi}}{{UAS - Snr1 - RNAi}};\frac{ + }{ + }$$

(all RNA_i_ lines provided by Vienna *Drosophila* Resource Center, Vienna, Austria).

#### Genetic modifier screen

Cell type-specific knockdown of *Snf5-related 1 (Snr1)* expression in glial cells was achieved by using the *repo-GAL4* driver and *UAS-Snr1-RNA*_*i*_, respectively. In order to establish a stable *Snr1* screening strain, transcriptional inhibitor *tubulin-GAL80* was used.

$$\begin{array}{ll}\frac{ + }{ + };\frac{{repo - GAL4,UAS - mCD8GFP,UAS - Snr1 - RNAi}}{{repo - GAL4,UAS - mCD8GFP,UAS - Snr1 - RNAi}};\\\frac{{repo - GAL4,UAS - mCD8GFP,}}{{tubulin - Gal80,\;TM6,\;Tb}}\end{array}$$

A genetic modifier screen was conducted by crossing the screening strain (decreased *Snr1* expression in glial cells) to RNA_i_ lines targeting a total of 59 cilia-associated *Drosophila* genes (Supplementary Table 8). Candidate genes for the modifier screen had been chosen based on a flybase (http://flybase.org) search for cilia-associated genes having at least one human orthologue. From the 59 genes examined, 45 (76%) genes can be found in the RNA Seq data generated in this study. Out of these, 27 (60%) and 22 (49%) genes show a similar trend towards downregulation upon *KIF3A* knockdown in BT-12 and CHLA-266 cells, respectively (Supplementary Fig. 7a, b), whereat 13 (29%) genes out of these show an overlap between both cell lines. Double knockdown of *Snr1* and the candidate genes was examined for the potential to mitigate deleterious effects of decreased *Snr1* and to rescue pupal lethality (positive shift). The shifting rates for animals affected by double knockdown were scored by counting the number of empty shells and comparing them with the whole number of pupae. Those candidate genes whose additional knockdown led to a pronounced positive shift (>20% hatching flies) were further validated in triplicate. To control for GAL4 dosage effects, the *Snr1* screening strain was crossed to *UAS-mCherry-RNA*_*i*_.

### Mice experiments

Six- to eight-week-old NOD.Cg-Prkdcscid Il2rgtm1Wjl/SzJ (NSG) mice were purchased from Charles River Laboratories France (Lyon, France). All animals were kept in specific pathogen-free (SPF) facilities, randomly housed per groups under standard conditions (at 20–22 °C under 10 h dark/14 h light), and given free access to food (RM3, SDS Diets, DIETEX, France) and water (decontaminated by reverse osmosis). In accordance with Directive 2010/63/EU (transposed to Portuguese legislation through Decreto-Lei No. 113/2013, of August 7th), all animal procedures were approved by the institutional animal welfare body (ORBEA-iMM), in order to ensure that the use of animals complies with all applicable legislation and follows the 3 R’s principle, and licensed by the Portuguese competent authority (Direcção Geral de Alimentação e Veterinária, license number: 012028\2016). Human endpoints were established for 10% body weight loss, paralysis and neurological impairment. Before any invasive procedure, mice were anesthetized using a mixture of 75 mg/kg BW ketamine and 1 mg/kg BW medetomidine. Mice were injected intracranially with BT-12 cells (2.5 × 10^5^ cells/3 µL, per mouse) in the right hemisphere of the frontal cortex. All mice were monitored for body weight, discomfort and distress every other day. Once any of the aforementioned human endpoints was reached, the mice were euthanized using anesthetic overdose with sodium pentobarbital, and the central neural system was collected for histopathologic analysis.

### Statistical analyses

Statistical analyses were performed using GraphPad Prism 5.03 (GraphPad Software, San Diego, CA, USA). All data are presented as mean ± SEM of at least three biologically independent experiments, unless stated otherwise. Comparisons between groups were made employing *t*-test, if not otherwise specified. *p*-values < 0.05 were considered significant. Dose-response curves were generated using Python (Python Software Foundation, Wilmington, NC, USA).

### Reporting summary

Further information on research design is available in the Nature Research Reporting Summary linked to this article.

## Supplementary information

Supplementary Material

Supplementary Figure 1

Supplementary Figure 2

Supplementary Figure 3

Supplementary Figure 4

Supplementary Figure 5

Supplementary Figure 6

Supplementary Figure 7

Supplementary Tables

Uncropped Western blots

Reporting Summary

## Data Availability

The datasets generated and analysed during the current study are available at NCBI GenBank (accession number: GSE179668) and PRIDE (dataset identifier: PXD027211).

## References

[1] Rorke LB, Packer RJ, Biegel JA (1996). Central nervous system atypical teratoid/rhabdoid tumors of infancy and childhood: Definition of an entity. J Neurosurg.

[2] Nesvick CL, Nageswara Rao AA, Raghunathan A, Biegel JA, Daniels DJ (2019). Case-based review: Atypical teratoid/rhabdoid tumor. Neurooncol Pr.

[3] Biegel JA, Zhou JY, Rorke LB, Stenstrom C, Wainwright LM, Fogelgren B (1999). Germ-line and acquired mutations of INI1 in atypical teratoid and rhabdoid tumors. Cancer Res.

[4] Hasselblatt M, Nagel I, Oyen F, Bartelheim K, Russell RB, Schuller U (2014). SMARCA4-mutated atypical teratoid/rhabdoid tumors are associated with inherited germline alterations and poor prognosis. Acta Neuropathol.

[5] Holdhof D, Johann PD, Spohn M, Bockmayr M, Safaei S, Joshi P (2021). Atypical teratoid/rhabdoid tumors (ATRTs) with SMARCA4 mutation are molecularly distinct from SMARCB1-deficient cases. Acta Neuropathol.

[6] Ginn KF, Gajjar A (2012). Atypical teratoid rhabdoid tumor: Current therapy and future directions. Front Oncol.

[7] Fruhwald MC, Biegel JA, Bourdeaut F, Roberts CW, Chi SN (2016). Atypical teratoid/rhabdoid tumors-current concepts, advances in biology, and potential future therapies. Neuro Oncol.

[8] Richardson EA, Ho B, Huang A (2018). Atypical teratoid rhabdoid tumour: From tumours to therapies. J Korean Neurosurg Soc.

[9] Kralik SF, Ho CY, Finke W, Buchsbaum JC, Haskins CP, Shih CS (2015). Radiation necrosis in pediatric patients with brain tumors treated with proton radiotherapy. AJNR Am J Neuroradiol.

[10] Torchia J, Golbourn B, Feng S, Ho KC, Sin-Chan P, Vasiljevic A (2016). Integrated (epi)-Genomic analyses identify subgroup-specific therapeutic targets in CNS rhabdoid tumors. Cancer Cell.

[11] Johann PD, Erkek S, Zapatka M, Kerl K, Buchhalter I, Hovestadt V (2016). Atypical Teratoid/Rhabdoid tumors are comprised of three epigenetic subgroups with distinct enhancer landscapes. Cancer Cell.

[12] Ho B, Johann PD, Grabovska Y, De Dieu Andrianteranagna MJ, Yao F, Fruhwald M (2020). Molecular subgrouping of atypical teratoid/rhabdoid tumors-a reinvestigation and current consensus. Neuro Oncol.

[13] Malicki JJ, Johnson CA (2017). The Cilium: Cellular antenna and central processing unit. Trends Cell Biol.

[14] Wheway G, Nazlamova L, Hancock JT (2018). Signaling through the Primary Cilium. Front Cell Dev Biol.

[15] Anvarian Z, Mykytyn K, Mukhopadhyay S, Pedersen LB, Christensen ST (2019). Cellular signalling by primary cilia in development, organ function and disease. Nat Rev Nephrol.

[16] Reiter JF, Leroux MR (2017). Genes and molecular pathways underpinning ciliopathies. Nat Rev Mol Cell Biol.

[17] Eguether T, Hahne M. Mixed signals from the cell’s antennae: Primary cilia in cancer. EMBO Rep. 2018;19:e46589. 10.15252/embr.201846589 PMC6216287 30348893

[18] Zingg D, Debbache J, Pena-Hernandez R, Antunes AT, Schaefer SM, Cheng PF (2018). EZH2-mediated primary cilium deconstruction drives metastatic melanoma formation. Cancer Cell.

[19] Egeberg DL, Lethan M, Manguso R, Schneider L, Awan A, Jorgensen TS (2012). Primary cilia and aberrant cell signaling in epithelial ovarian cancer. Cilia.

[20] Emoto K, Masugi Y, Yamazaki K, Effendi K, Tsujikawa H, Tanabe M (2014). Presence of primary cilia in cancer cells correlates with prognosis of pancreatic ductal adenocarcinoma. Hum Pathol.

[21] Dvorak J, Hadzi Nikolov D, Dusek L, Filipova A, Richter I, Buka D (2016). Prognostic significance of the frequency of primary cilia in cells of small bowel and colorectal adenocarcinoma. J BUON.

[22] Han YG, Kim HJ, Dlugosz AA, Ellison DW, Gilbertson RJ, Alvarez-Buylla A (2009). Dual and opposing roles of primary cilia in medulloblastoma development. Nat Med.

[23] Sarkisian MR, Siebzehnrubl D, Hoang-Minh L, Deleyrolle L, Silver DJ, Siebzehnrubl FA (2014). Detection of primary cilia in human glioblastoma. J Neurooncol.

[24] Li L, Grausam KB, Wang J, Lun MP, Ohli J, Lidov HG (2016). Sonic Hedgehog promotes proliferation of Notch-dependent monociliated choroid plexus tumour cells. Nat Cell Biol.

[25] Mirvis M, Stearns T, James Nelson W (2018). Cilium structure, assembly, and disassembly regulated by the cytoskeleton. Biochem J.

[26] Firestone AJ, Weinger JS, Maldonado M, Barlan K, Langston LD, O’Donnell M (2012). Small-molecule inhibitors of the AAA+ ATPase motor cytoplasmic dynein. Nature.

[27] Fish EN, Platanias LC (2014). Interferon receptor signaling in malignancy: a network of cellular pathways defining biological outcomes. Mol Cancer Res.

[28] Jeibmann A, Eikmeier K, Linge A, Kool M, Koos B, Schulz J (2014). Identification of genes involved in the biology of atypical teratoid/rhabdoid tumours using Drosophila melanogaster. Nat Commun.

[29] Liu H, Kiseleva AA, Golemis EA (2018). Ciliary signalling in cancer. Nat Rev Cancer.

[30] Huangfu D, Liu A, Rakeman AS, Murcia NS, Niswander L, Anderson KV (2003). Hedgehog signalling in the mouse requires intraflagellar transport proteins. Nature.

[31] Rohatgi R, Scott MP (2007). Patching the gaps in Hedgehog signalling. Nat Cell Biol.

[32] Wong SY, Seol AD, So PL, Ermilov AN, Bichakjian CK, Epstein EH (2009). Primary cilia can both mediate and suppress Hedgehog pathway-dependent tumorigenesis. Nat Med.

[33] Barakat MT, Humke EW, Scott MP (2013). Kif3a is necessary for initiation and maintenance of medulloblastoma. Carcinogenesis.

[34] Hoang-Minh LB, Deleyrolle LP, Siebzehnrubl D, Ugartemendia G, Futch H, Griffith B (2016). Disruption of KIF3A in patient-derived glioblastoma cells: Effects on ciliogenesis, hedgehog sensitivity, and tumorigenesis. Oncotarget.

[35] Youn YH, Han YG (2018). Primary Cilia in Brain Development and Diseases. Am J Pathol.

[36] Archer TC, Ehrenberger T, Mundt F, Gold MP, Krug K, Mah CK (2018). Proteomics, Post-translational Modifications, and Integrative Analyses Reveal Molecular Heterogeneity within Medulloblastoma Subgroups. Cancer Cell.

[37] Forget A, Martignetti L, Puget S, Calzone L, Brabetz S, Picard D (2018). Aberrant ERBB4-SRC Signaling as a Hallmark of Group 4 Medulloblastoma Revealed by Integrative Phosphoproteomic Profiling. Cancer Cell.

[38] Mun DG, Bhin J, Kim S, Kim H, Jung JH, Jung Y (2019). Proteogenomic characterization of human early-onset gastric cancer. Cancer Cell.

[39] Rivero-Hinojosa S, Lau LS, Stampar M, Staal J, Zhang H, Gordish-Dressman H (2018). Proteomic analysis of Medulloblastoma reveals functional biology with translational potential. Acta Neuropathol Commun.

[40] Sinha A, Huang V, Livingstone J, Wang J, Fox NS, Kurganovs N (2019). The Proteogenomic Landscape of Curable Prostate Cancer. Cancer Cell.

[41] Gordziel C, Bratsch J, Moriggl R, Knosel T, Friedrich K (2013). Both STAT1 and STAT3 are favourable prognostic determinants in colorectal carcinoma. Br J Cancer.

[42] Sun Y, Yang S, Sun N, Chen J (2014). Differential expression of STAT1 and p21 proteins predicts pancreatic cancer progression and prognosis. Pancreas.

[43] Takahashi A, Nakayama R, Ishibashi N, Doi A, Ichinohe R, Ikuyo Y (2014). Analysis of gene expression profiles of soft tissue sarcoma using a combination of knowledge-based filtering with integration of multiple statistics. PLoS One.

[44] Osborn JL, Greer SF (2015). Metastatic melanoma cells evade immune detection by silencing STAT1. Int J Mol Sci.

[45] Dimco G, Knight RA, Latchman DS, Stephanou A (2010). STAT1 interacts directly with cyclin D1/Cdk4 and mediates cell cycle arrest. Cell Cycle.

[46] Chin YE, Kitagawa M, Kuida K, Flavell RA, Fu XY (1997). Activation of the STAT signaling pathway can cause expression of caspase 1 and apoptosis. Mol Cell Biol.

[47] Miura Y, Tsujioka T, Nishimura Y, Sakaguchi H, Maeda M, Hayashi H (2006). TRAIL expression up-regulated by interferon-gamma via phosphorylation of STAT1 induces myeloma cell death. Anticancer Res.

[48] Chun HE, Johann PD, Milne K, Zapatka M, Buellesbach A, Ishaque N (2019). Identification and analyses of extra-cranial and cranial rhabdoid tumor molecular subgroups reveal tumors with cytotoxic T cell infiltration. Cell Rep.

[49] Hannus M, Beitzinger M, Engelmann JC, Weickert MT, Spang R, Hannus S (2014). siPools: Highly complex but accurately defined siRNA pools eliminate off-target effects. Nucleic Acids Res.

[50] Bradford MM (1976). A rapid and sensitive method for the quantitation of microgram quantities of protein utilizing the principle of protein-dye binding. Anal Biochem.

[51] Poschmann G, Seyfarth K, Besong Agbo D, Klafki HW, Rozman J, Wurst W (2014). High-fat diet induced isoform changes of the Parkinson’s disease protein DJ-1. J Proteome Res.

[52] Perez-Riverol Y, Csordas A, Bai J, Bernal-Llinares M, Hewapathirana S, Kundu DJ (2019). The PRIDE database and related tools and resources in 2019: improving support for quantification data. Nucleic Acids Res.

