# Supplementary material for: Primary cilia contribute to the aggressiveness of atypical teratoid/rhabdoid tumors
Source: Cell Death Dis. 2022 Sep 20;13(9):806. doi: 10.1038/s41419-022-05243-4 (PMC9489777; doi:10.1038/s41419-022-05243-4)
Supplement: Supplementary file 1 — Supplementary Material [file 41419_2022_5243_MOESM1_ESM.docx]

**Summary**

The Supplementary Material contains Supplementary Figure legends.

**Supplementary Figure legends**

**Supplementary Figure 1: Expression levels of *kinesin family member 3A* (*KIF3A*) in AT/RT cell lines.** (a) *KIF3A* expression levels were derived from RNA sequencing data. It is indicated if the cells are grown with (+) or without (-) fetal bovine serum (FBS). (b) Representative immunofluorescence images of AT/RT cell lines(1-5) showing stainings for pericentrin (green) and acetylated tubulin (red), detecting the basal body and the axoneme of the primary cilium, respectively. Nuclei were counterstained with 4´,6‐diamidin‐2‐phenylindol (DAPI, blue). Arrows indicate primary cilia. The scale bar corresponds to 10 µm.

**Supplementary Figure 2: Knockdown of *kinesin family member 3A* (*KIF3A*) disrupts primary ciliogenesis in the AT/RT cell lines BT‐12 and CHLA‐266.** Transient knockdown of *KIF3A* was achieved using siPOOLs. Stable knockdown of *KIF3A* was achieved using shRNA‐based (BT‐12) or CRISPRi‐based (CHLA‐266) technology. (a) mRNA expression was normalized to the housekeeping genes *glyceraldehyde 3‐phosphate dehydrogenase* (*GAPDH*) and *phosphoglycerate kinase 1* (*PGK1*) and calculated relative to the CTRL. (b) Shown are representative Western blot images for KIF3A and actin as a loading control following transient and stable *KIF3A* knockdown. (c) Quantification of the percentage of ciliated cells revealed reduction of ciliated cells following *KIF3A* knockdown in BT‐12 and CHLA‐266 cells. Representative immunofluorescence images showing stainings for Pericentrin (green) and Acetylated tubulin (red), detecting the basal body and the axoneme of the primary cilium, respectively, are shown in (d). Nuclei were counterstained with 4´,6‐diamidin‐2‐phenylindol (DAPI, blue). Arrows indicate either primary cilia in the control cells or disrupted primary ciliogenesis in the *KIF3A* knockdown cells. Values shown represent mean ±SEM of three biologically independent replicates. ns: not significant; *p<0.05; **p<0.01; ***p<0.001 (t‐test). CTRL: siCTRL/shCTRL/CRISPRiCTRL; KD: knockdown. The scale bar corresponds to 10 μm.

**Supplementary Figure 3: Ciliobrevin D (CilioD) treatment disrupts primary ciliogenesis in the AT/RT cell lines BT‐12 and CHLA‐266.** BT‐12 and CHLA‐266 cells were seeded in complete medium. The next day, complete medium was replaced with medium lacking serum and the cells were treated with 30 µM CilioD or dimethylsulfoxide (DMSO) as a negative control. (a-b) For the analysis of the dose‐dependent response to CilioD, BT‐12 and CHLA‐266 cells were seeded on white 384‐well plates and CilioD was added in a concentration gradient (0.005 µM ‐ 100 µM) in a randomized manner using the D300(e) Digital Dispenser from Tecan. The DMSO concentration was normalized to 0.25% in each well. After 72 h, cell viability was analyzed. Values shown represent mean ±SD of three replicates. (c) Immunofluorescence confirmed that serum starvation increases the percentage of ciliated cells in BT‐12 and CHLA‐266 cells. Representative immunofluorescence images are shown in (d). (e) Immunofluorescence demonstrated complete loss of ciliated cells in BT‐12 and CHLA‐266 cells. Representative immunofluorescence images are shown in (f). Quantitative real‐time PCR revealed reduced expression of *glioma‐associated oncogene 1* (*GLI1*), *patched 1* (*PTCH1*) and *smoothened* (*SMO*) following CilioD treatment in BT‐12 (g) and CHLA‐266 (h) cells. mRNA expression was normalized to the housekeeping genes *glyceraldehyde 3‐phosphate dehydrogenase* (*GAPDH*) and *phosphoglycerate kinase 1* (*PGK1*) and calculated relative to DMSO. Values shown represent mean ±SEM of three biologically independent replicates. nd: not detected; **p<0.01; ***p<0.001 (t‐test).

**Supplementary Figure 4: Interferon-induced genes are upregulated following *kinesin family member 3A* (*KIF3A*) knockdown in the AT/RT cell lines BT‐12 and CHLA‐266.** siRNA‐mediated knockdown of *KIF3A* in BT‐12 (a) and CHLA‐266 (b) cells was achieved using siPOOLs. mRNA expression was normalized to the housekeeping genes *glyceraldehyde 3‐phosphate dehydrogenase* (*GAPDH*) and *phosphoglycerate kinase 1* (*PGK1*) and calculated relative to siCTRL. Values shown represent mean ± SEM of three biologically independent replicates. *p<0.05; **p<0.01; ***p<0.001 (t‐test).

**Supplementary Figure 5: Interferon‐induced genes show anti‐correlated expression with *kinesin family member 3A* (*KIF3A*) in AT/RT tumor samples.** Gene expression data are derived from the Tumor ATRT ‐ Kool ‐ 49 ‐ MAS5.0 ‐ u133p2 data set available at the R2: Genomics analysis and visualization platform (http://r2.amc.nl).

**Supplementary Figure 6: Comparison between two high-throughput screens of genes functionally involved in the lethal phenotype of *Snf5‐related 1* (*Snr1*) deficiency.** The proportion of cilia‐associated genes causing a positive shift of the phenotype in this study (genes associated with ciliogenesis) was significantly higher compared to the proportion previously reported(6) for a large set of genes with known nervous system expression. (14/59 (23.7%) vs 60/1015 (5.9%); Chi‐square: 27.592; df:1, ***p<0.001).

**Supplementary Figure 7: Unsupervised hierarchical clustering of cilia-associated genes expressed following *kinesin family member 3A (KIF3A)* knockdown in the AT/RT cell lines BT‐12 and CHLA‐266.** The heatmaps for both BT-12 (a) and CHLA-266 (b) cells are based on the cilia-associated genes evaluated for the modifier screen in *Drosophila* (Supplementary Table 8). Upregulated genes are shown in red and downregulated genes are shown in green.

**References**

1. Theruvath J, Sotillo E, Mount CW, Graef CM, Delaidelli A, Heitzeneder S*, et al.* Locoregionally administered B7-H3-targeted CAR T cells for treatment of atypical teratoid/rhabdoid tumors. Nat Med 2020;26:712-9

2. Erdreich-Epstein A, Robison N, Ren X, Zhou H, Xu J, Davidson TB*, et al.* PID1 (NYGGF4), a new growth-inhibitory gene in embryonal brain tumors and gliomas. Clin Cancer Res 2014;20:827-36

3. D'Cunja J, Shalaby T, Rivera P, von Buren A, Patti R, Heppner FL*, et al.* Antisense treatment of IGF-IR induces apoptosis and enhances chemosensitivity in central nervous system atypical teratoid/rhabdoid tumours cells. Eur J Cancer 2007;43:1581-9

4. Xu J, Margol A, Asgharzadeh S, Erdreich-Epstein A. Pediatric brain tumor cell lines. J Cell Biochem 2015;116:218-24

5. Bartl J, Zanini M, Bernardi F, Forget A, Blumel L, Talbot J*, et al.* The HHIP-AS1 lncRNA promotes tumorigenicity through stabilization of dynein complex 1 in human SHH-driven tumors. Nat Commun 2022;13:4061

6. Jeibmann A, Eikmeier K, Linge A, Kool M, Koos B, Schulz J*, et al.* Identification of genes involved in the biology of atypical teratoid/rhabdoid tumours using Drosophila melanogaster. Nat Commun 2014;5:4005
