# Supplementary figures and images for: Primary cilia contribute to the aggressiveness of atypical teratoid/rhabdoid tumors
Source: Cell Death Dis. 2022 Sep 20;13(9):806. doi: 10.1038/s41419-022-05243-4 (PMC9489777; doi:10.1038/s41419-022-05243-4)

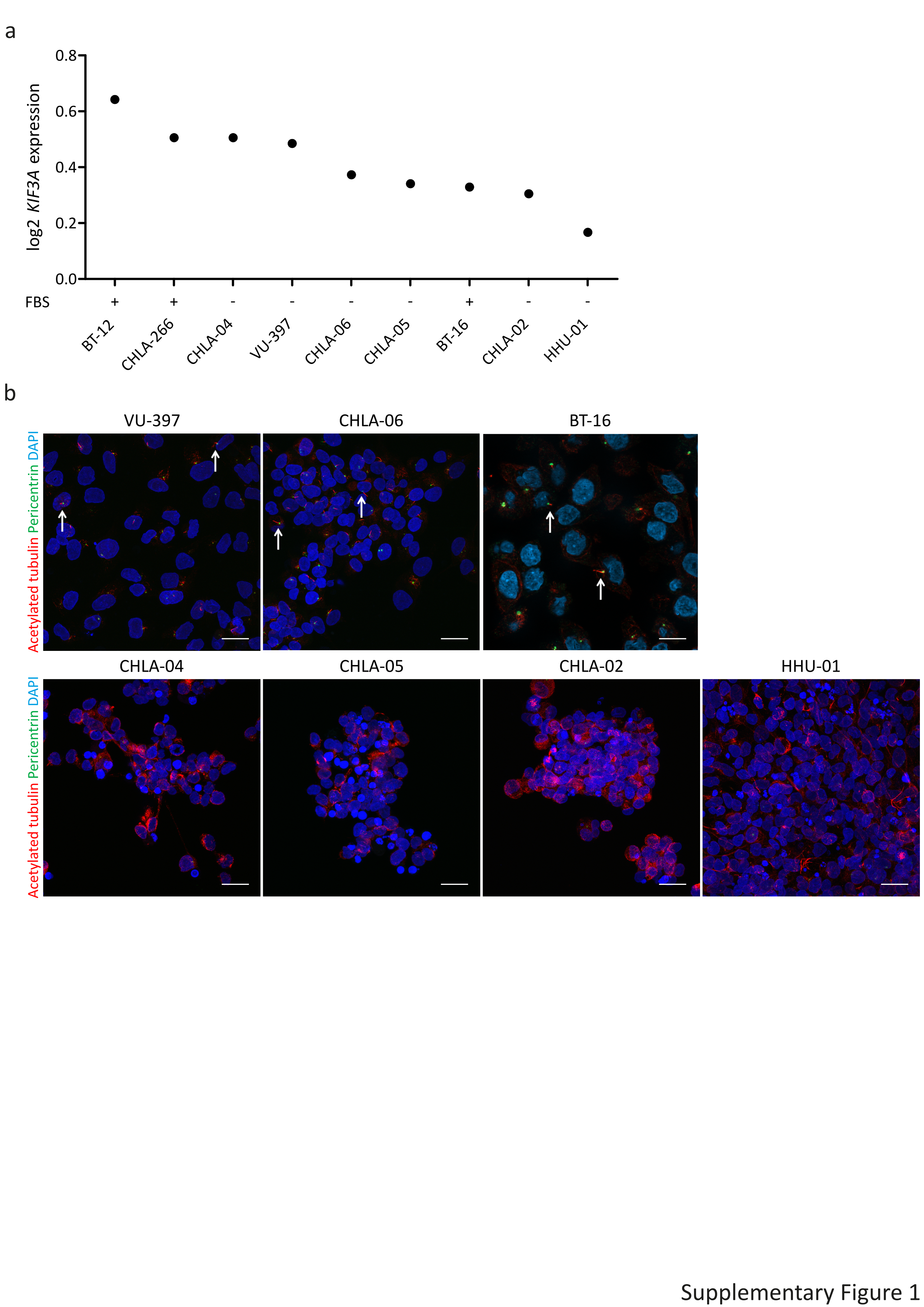

Supplement: Supplementary file 2 — Supplementary Figure 1 [file 41419_2022_5243_MOESM2_ESM.png]

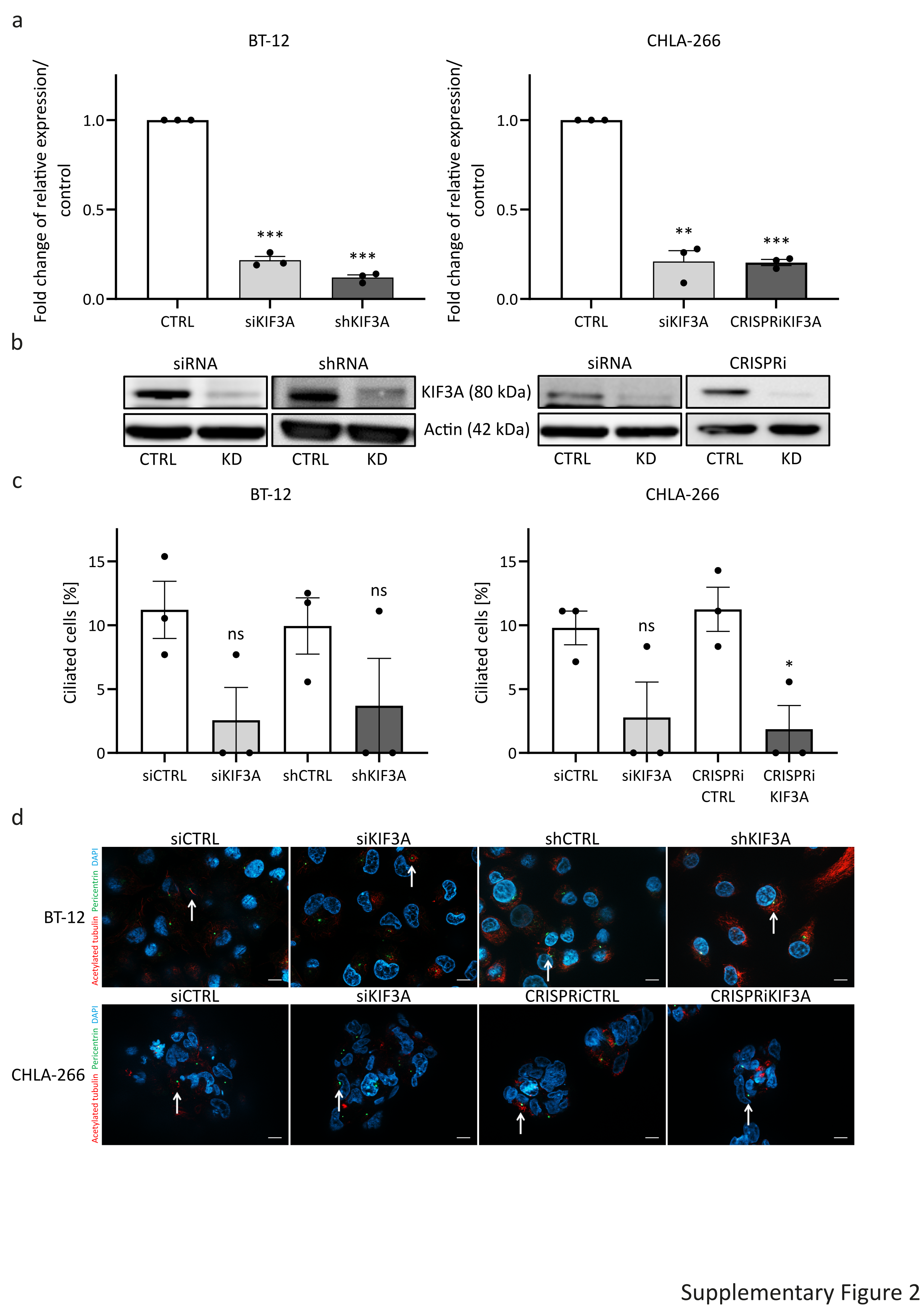

Supplement: Supplementary file 3 — Supplementary Figure 2 [file 41419_2022_5243_MOESM3_ESM.png]

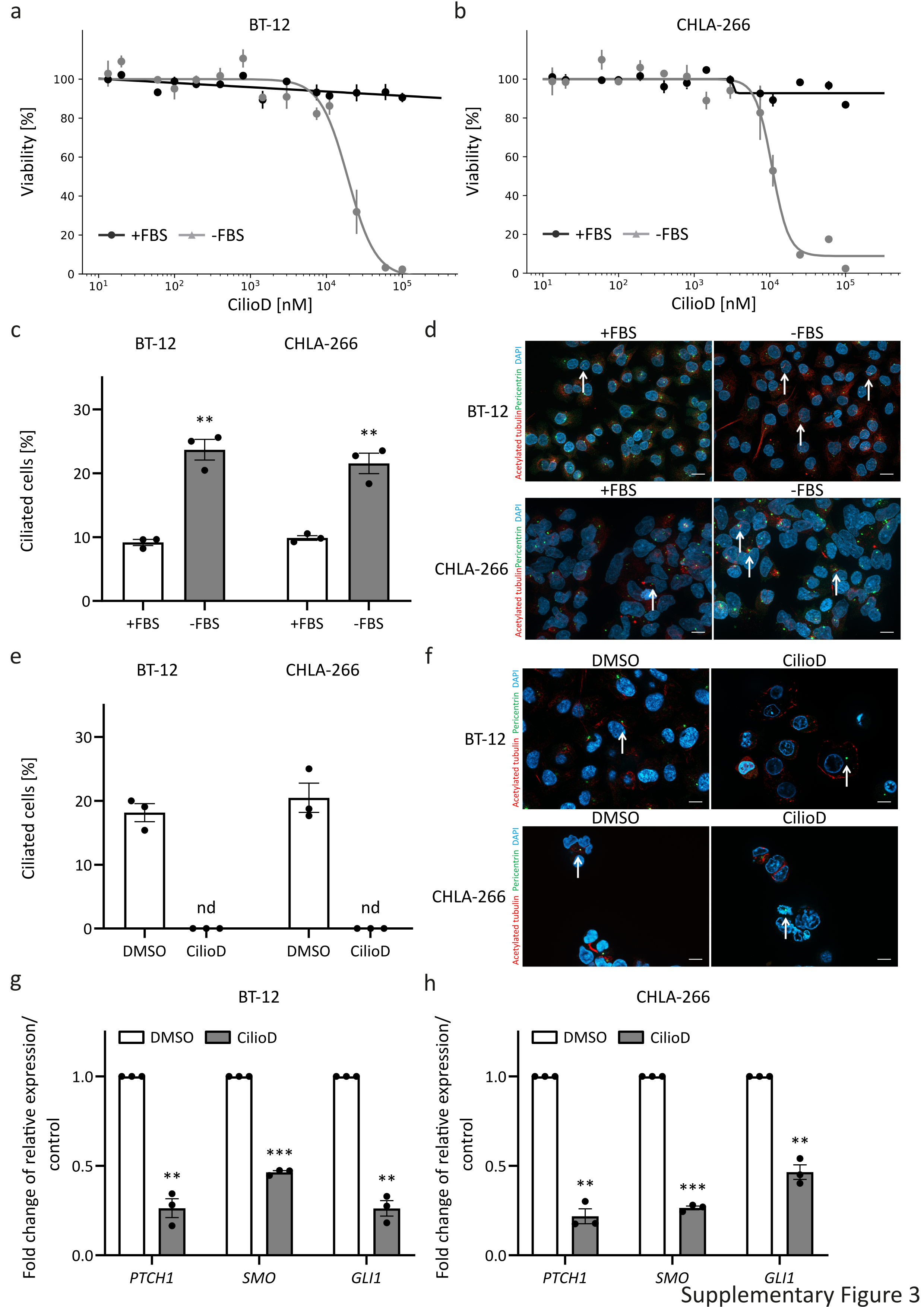

Supplement: Supplementary file 4 — Supplementary Figure 3 [file 41419_2022_5243_MOESM4_ESM.png]

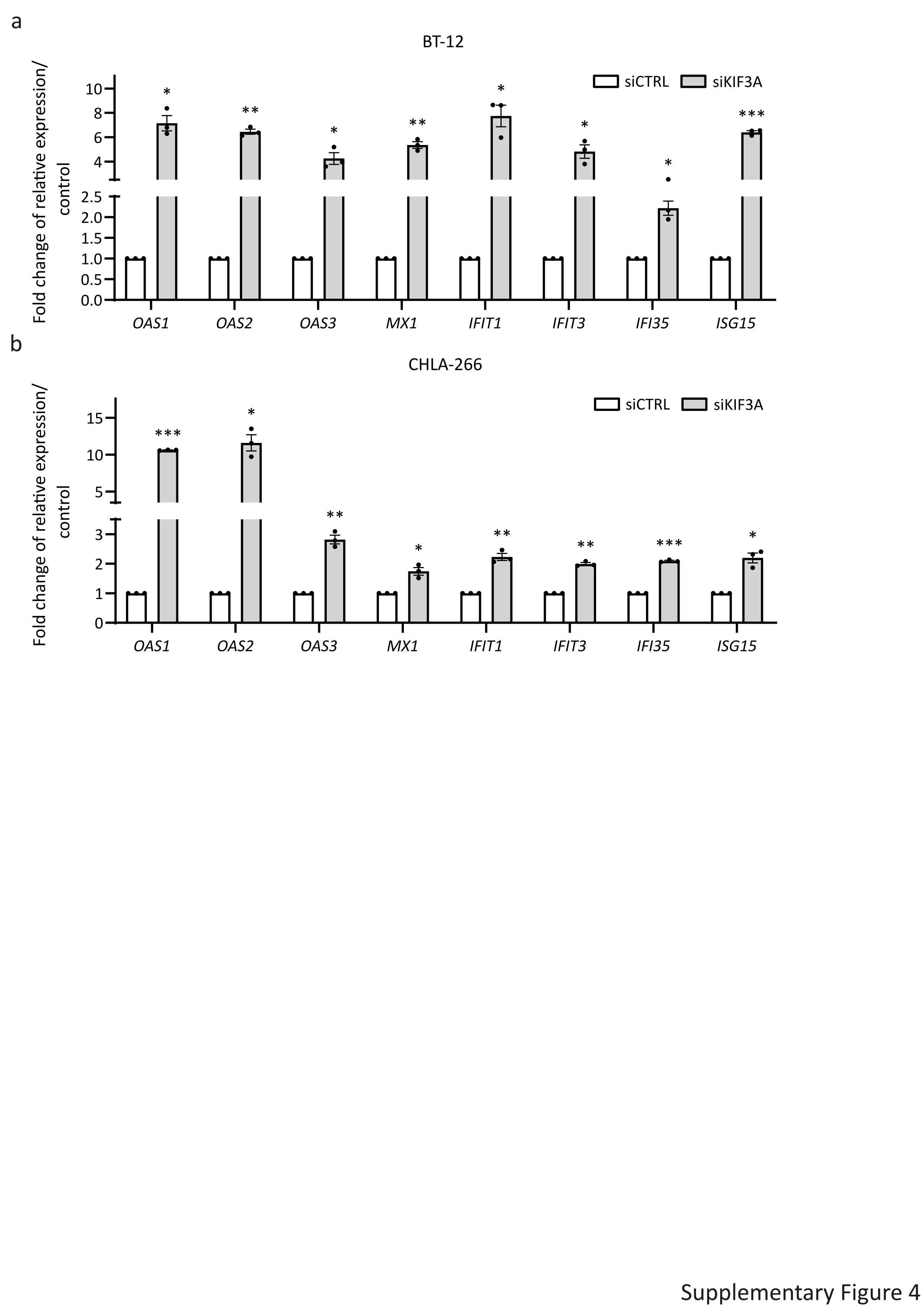

Supplement: Supplementary file 5 — Supplementary Figure 4 [file 41419_2022_5243_MOESM5_ESM.png]

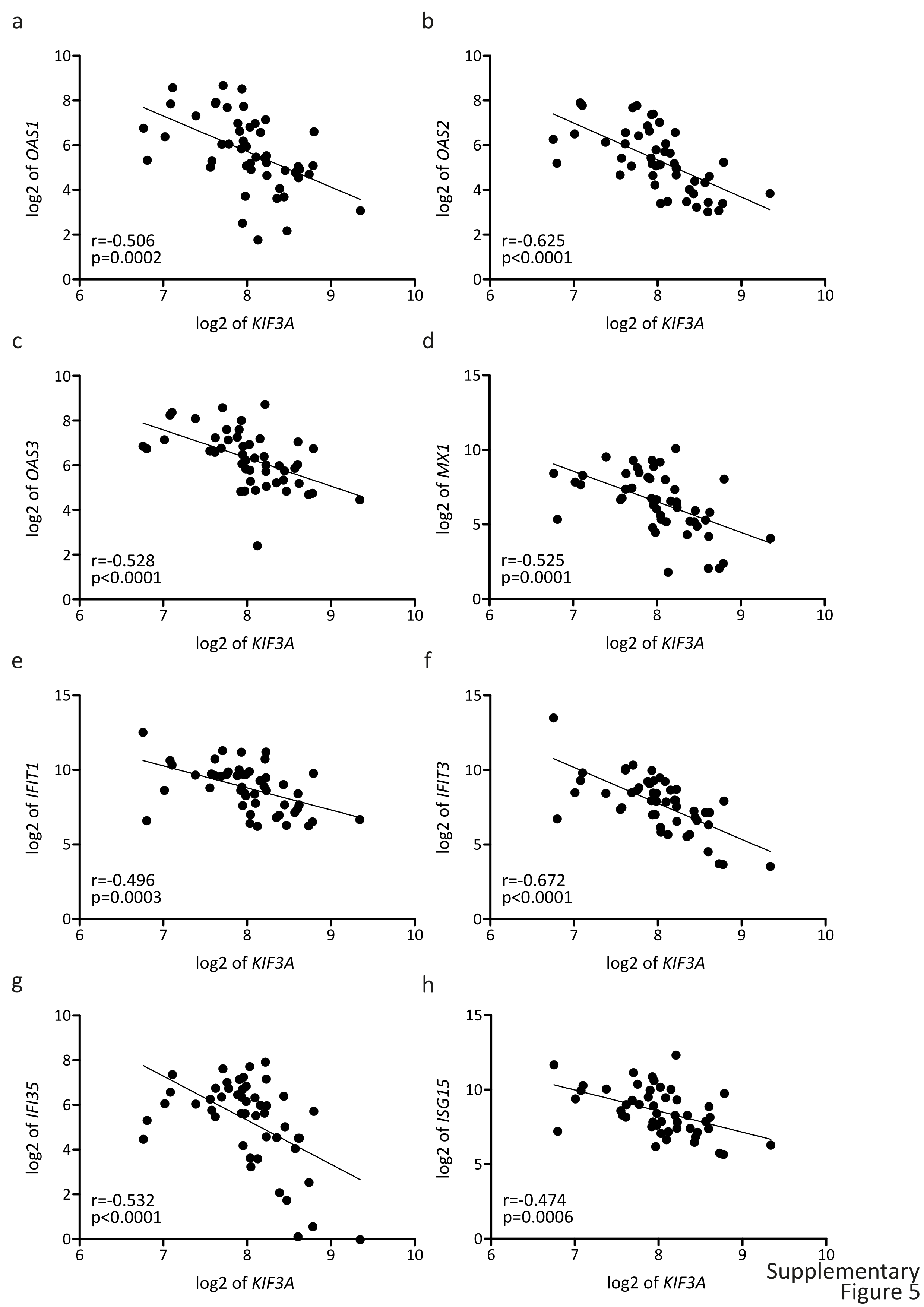

Supplement: Supplementary file 6 — Supplementary Figure 5 [file 41419_2022_5243_MOESM6_ESM.png]

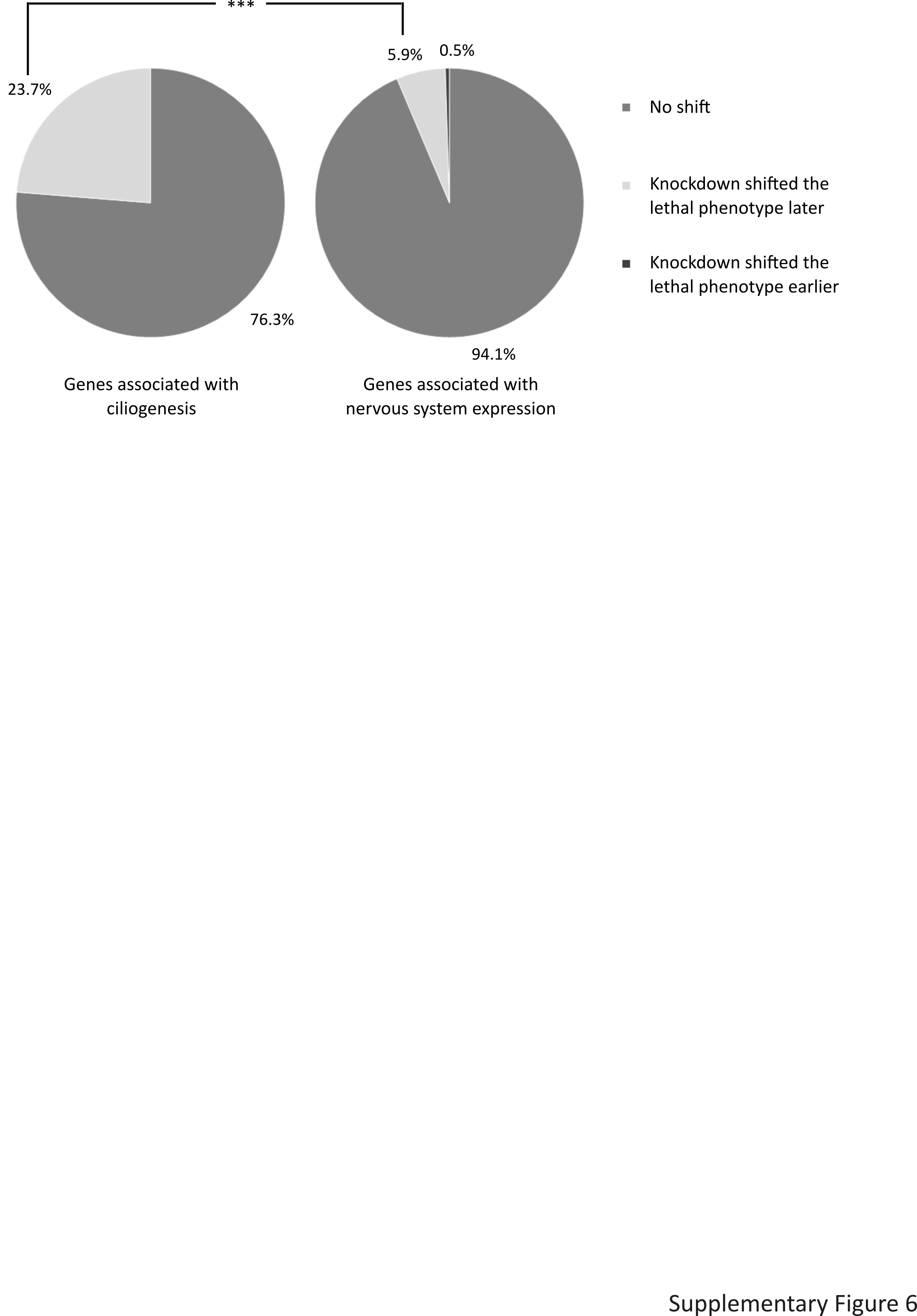

Supplement: Supplementary file 7 — Supplementary Figure 6 [file 41419_2022_5243_MOESM7_ESM.png]

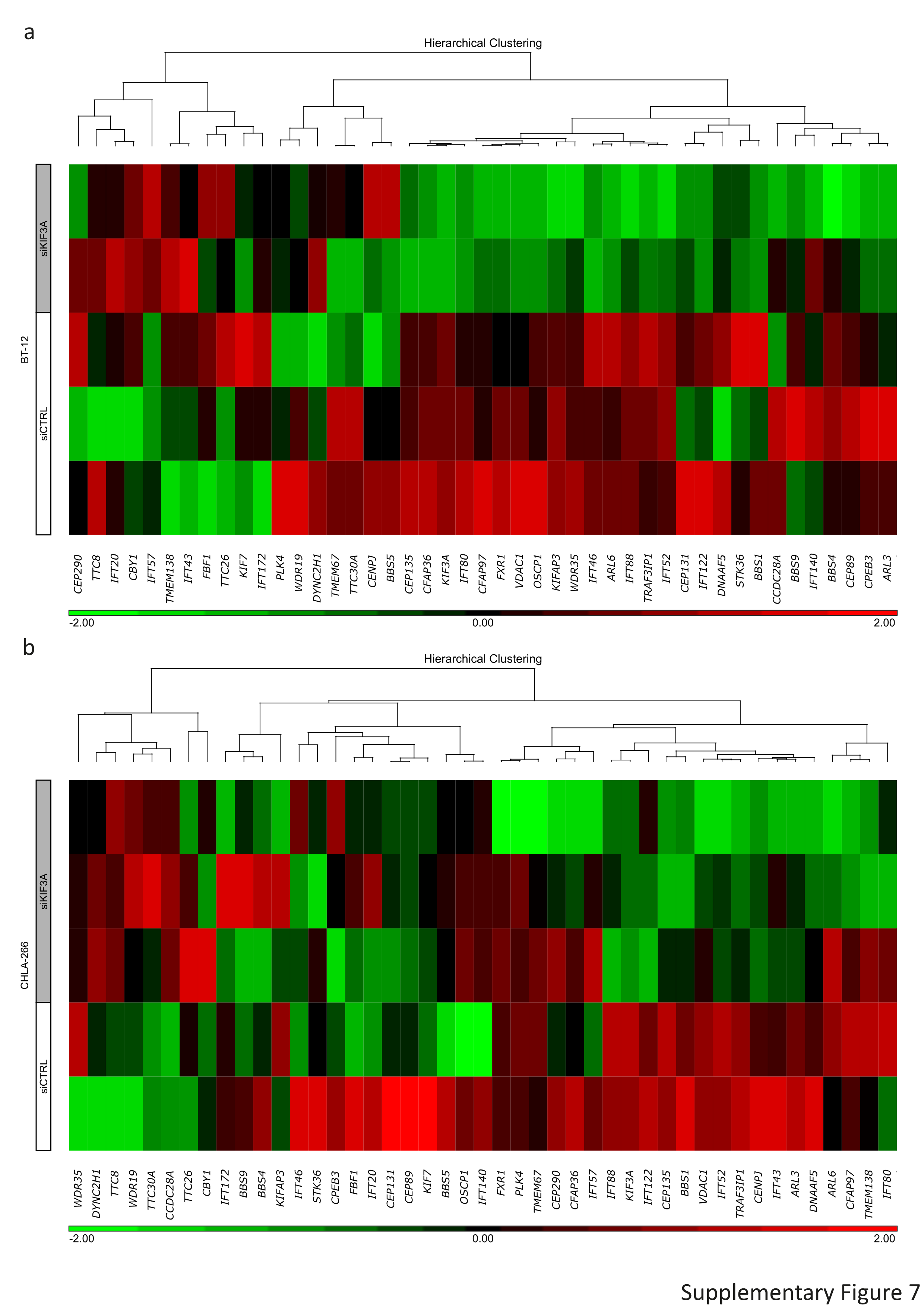

Supplement: Supplementary file 8 — Supplementary Figure 7 [file 41419_2022_5243_MOESM8_ESM.png]

**Uncropped Western blots**

**Figure 5**

**
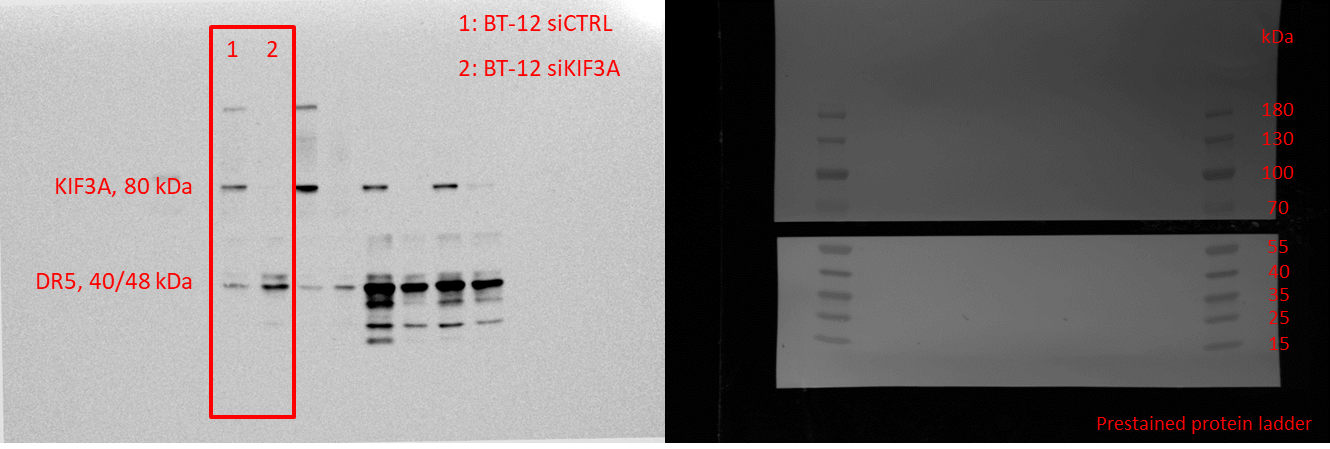
**

**
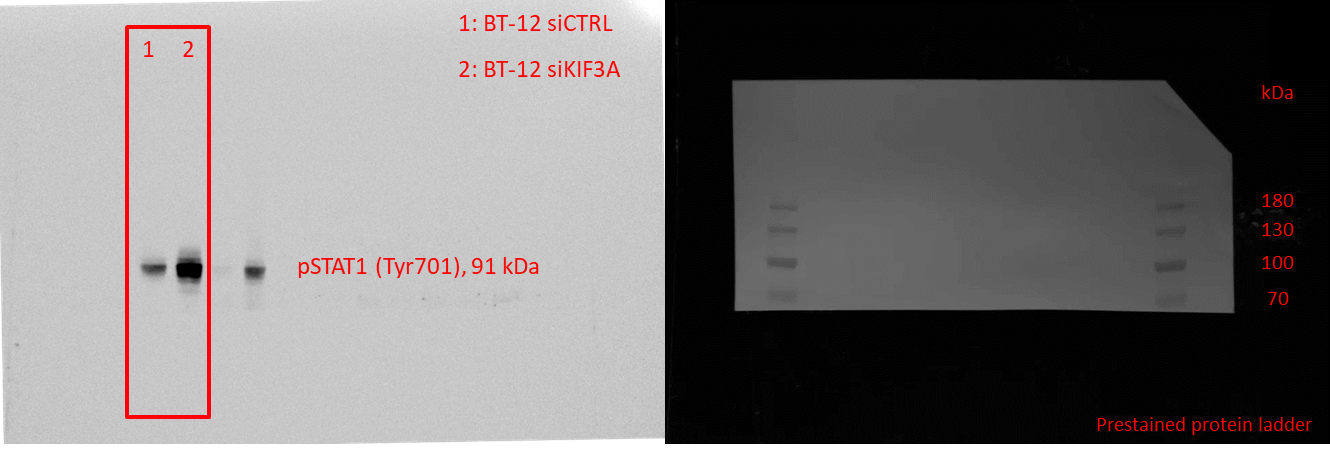
**

**
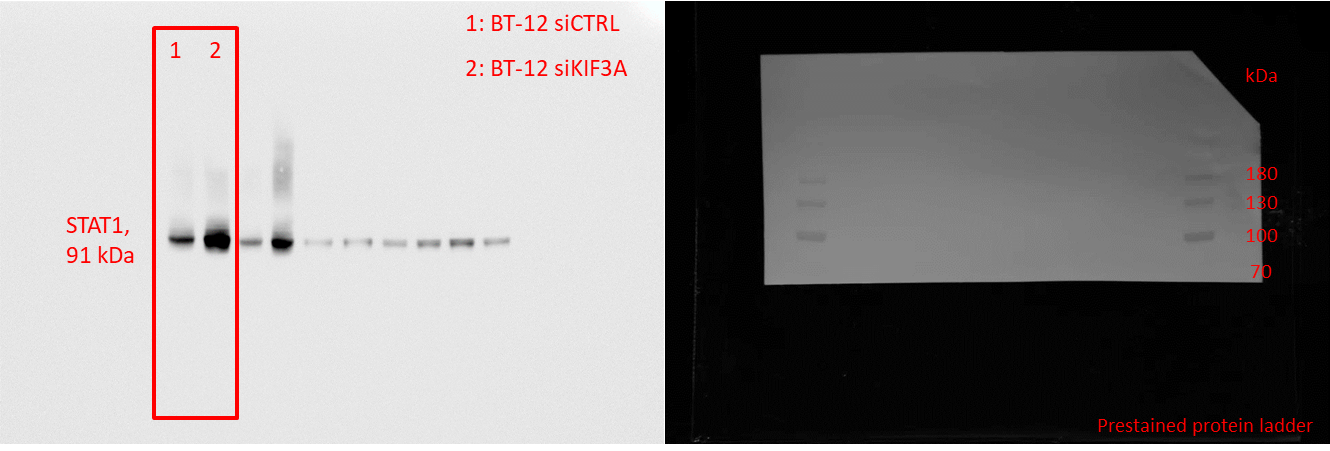
**

**
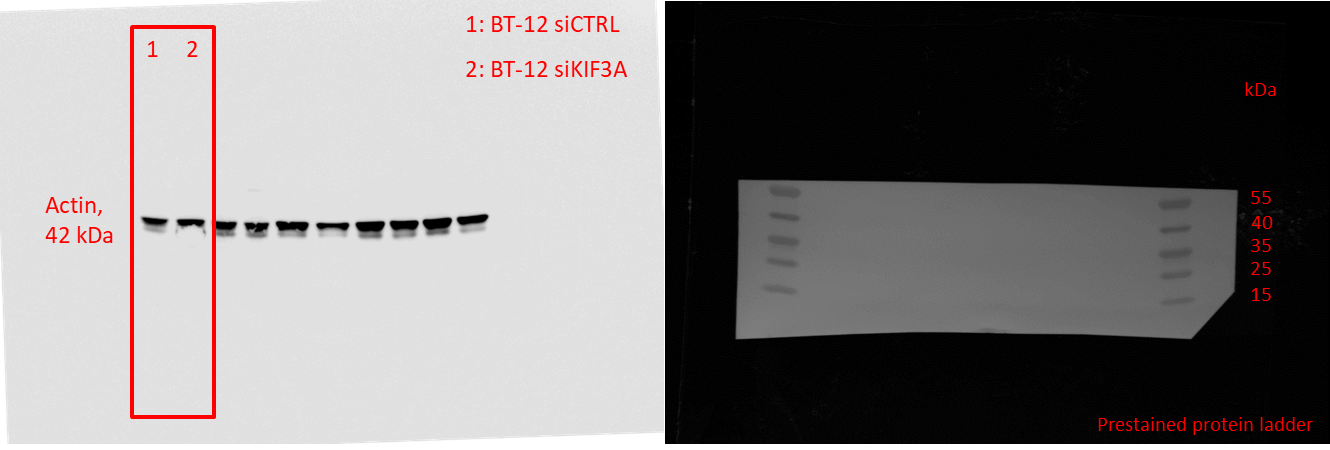
**

**
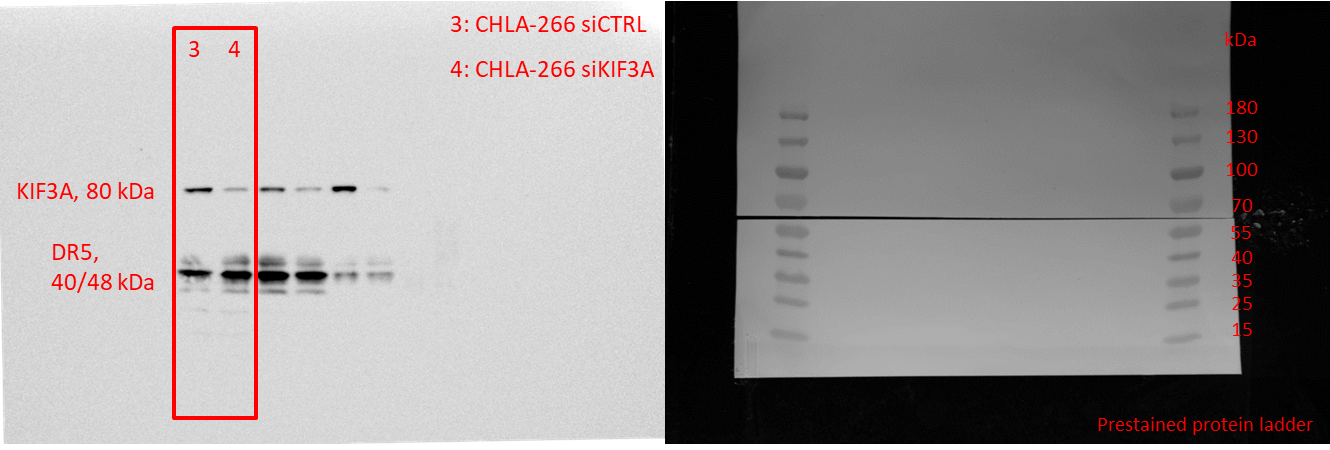
**

**
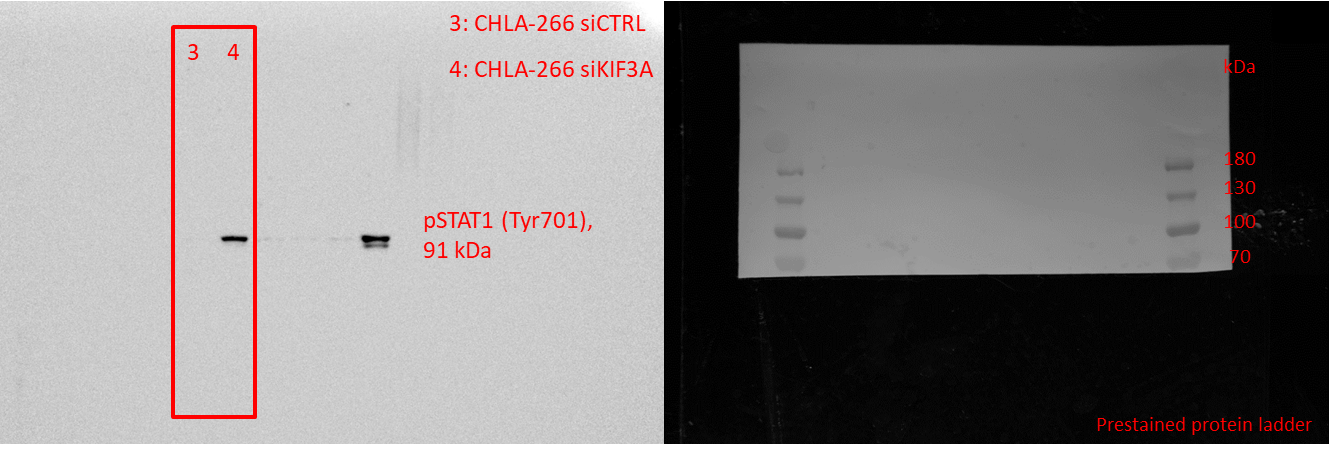
**

**
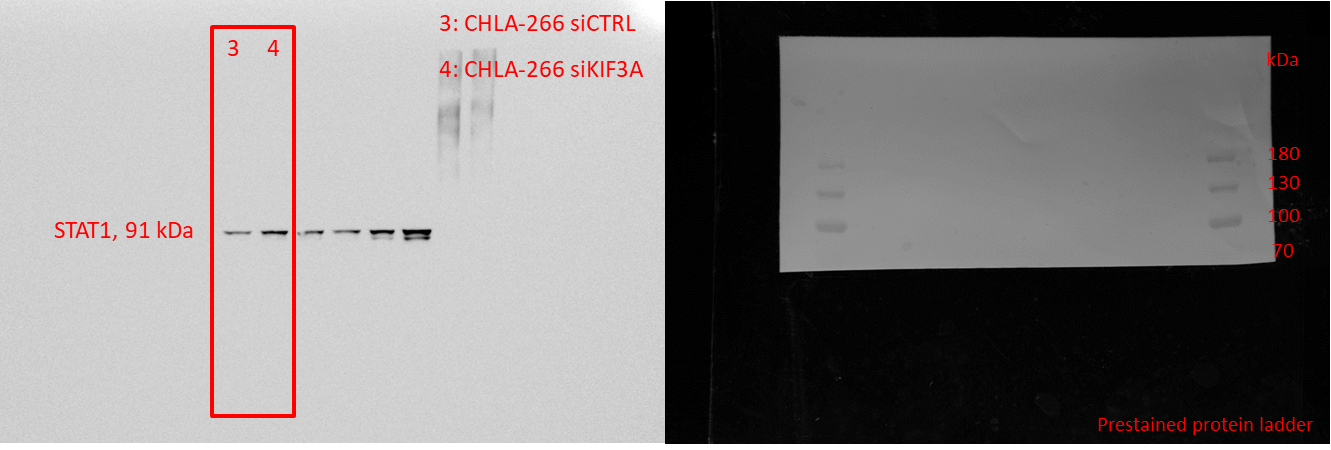
**

**
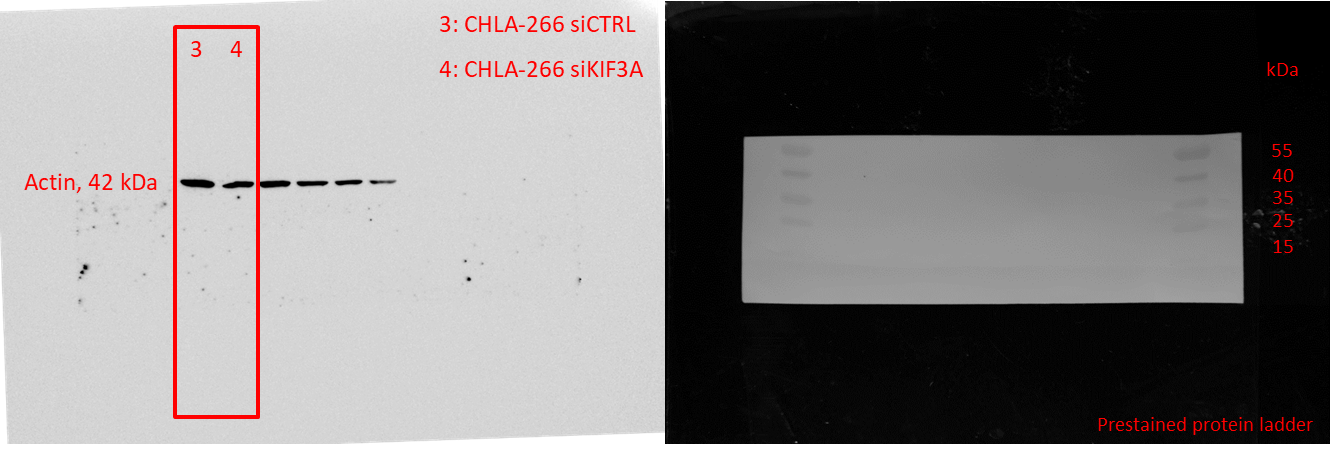
**

**Supplementary Figure 2**

**
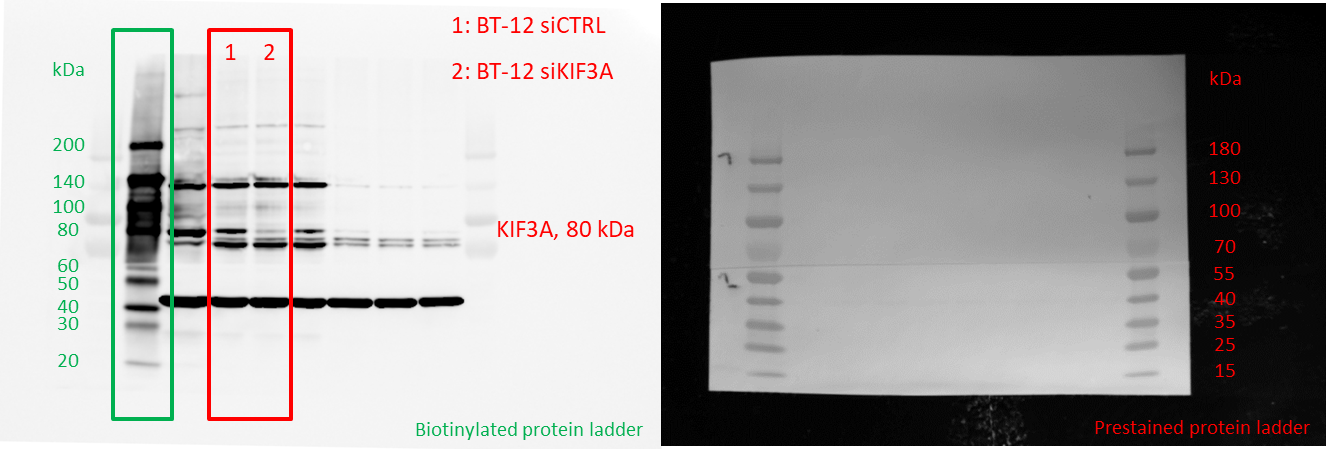
**

**
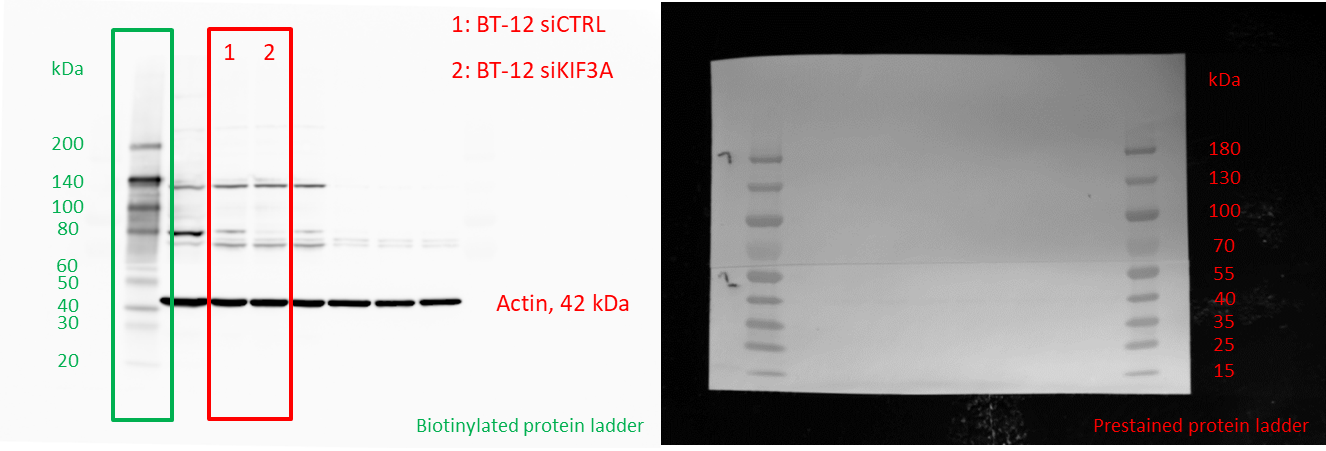
**

**
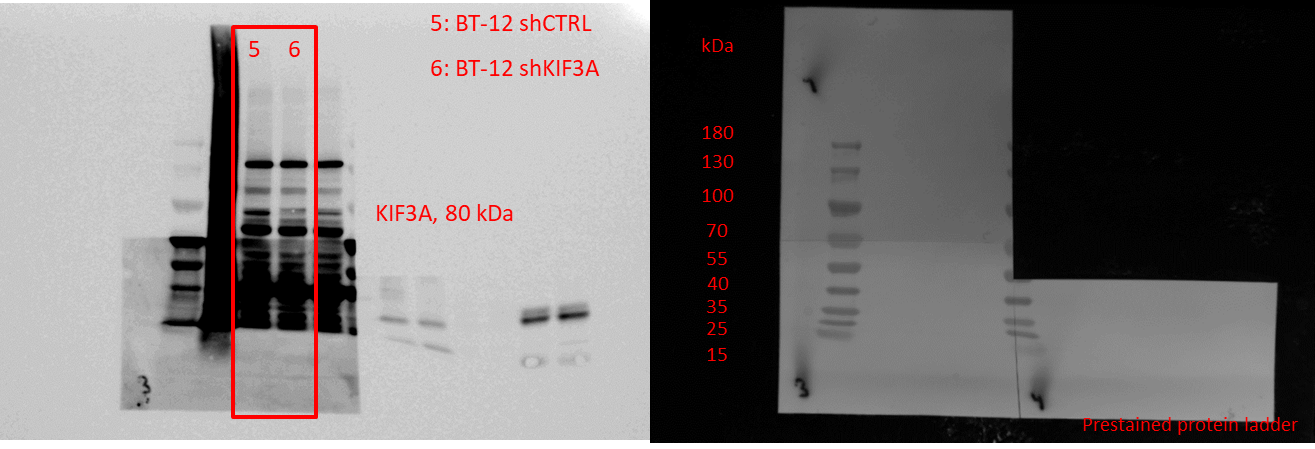
**

**
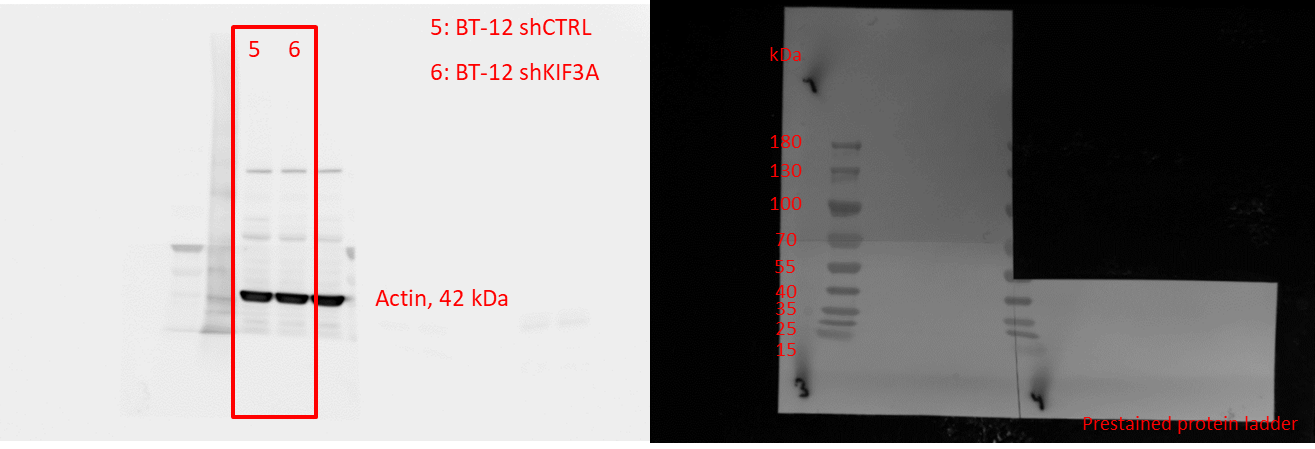
**

**
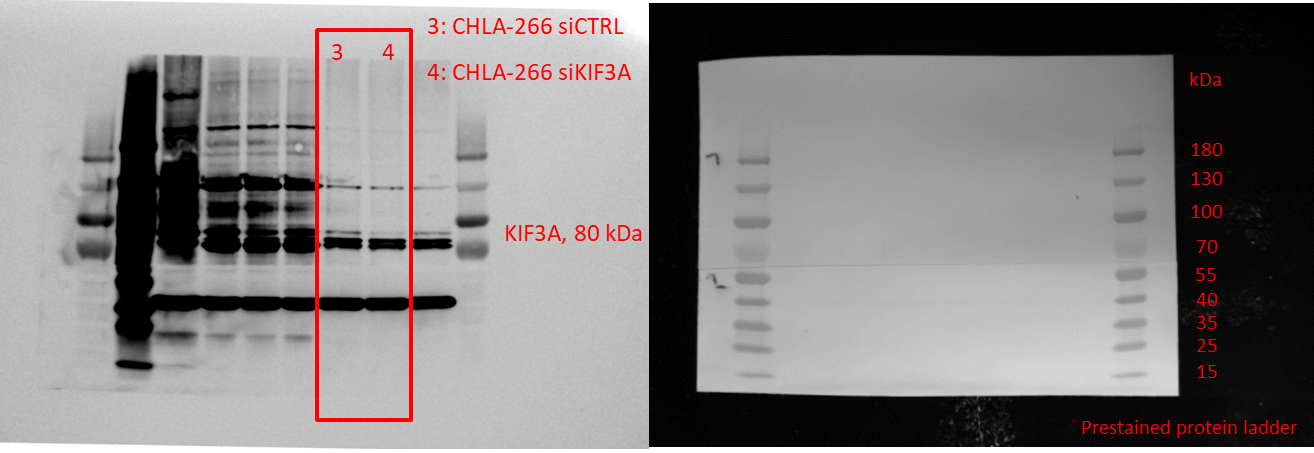
**

**
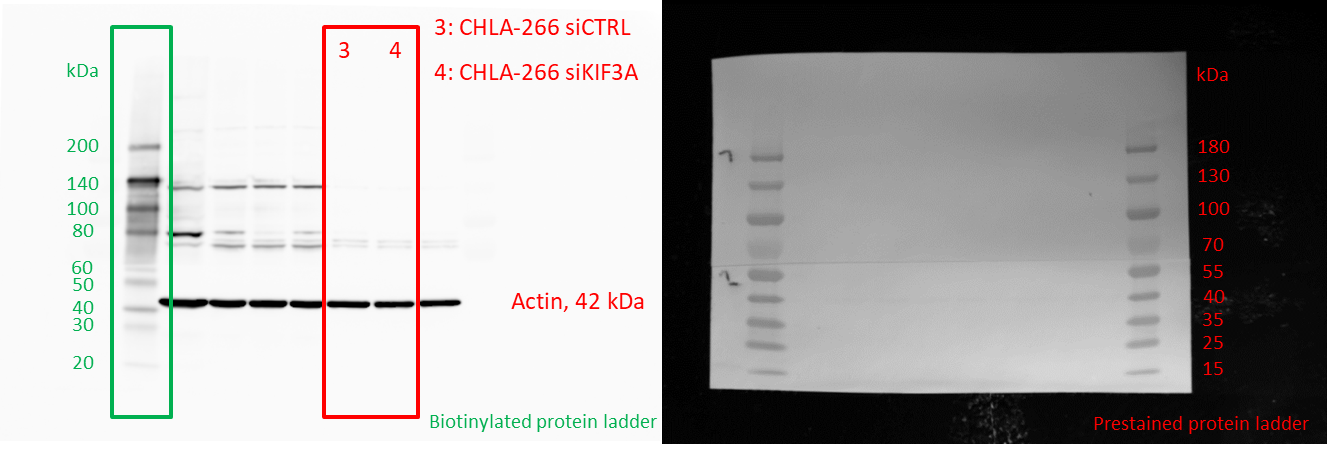
**

**
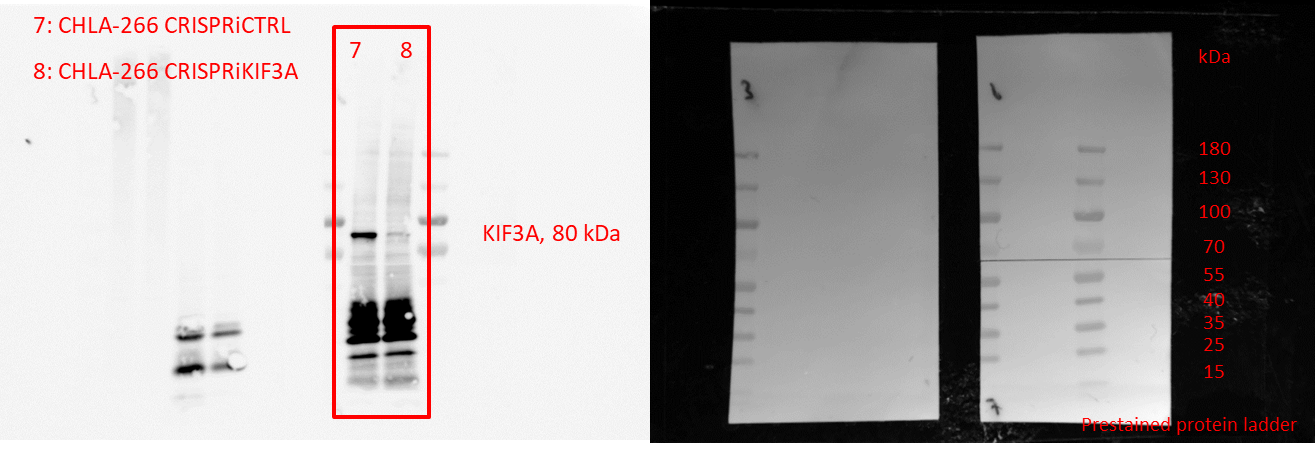
**

**
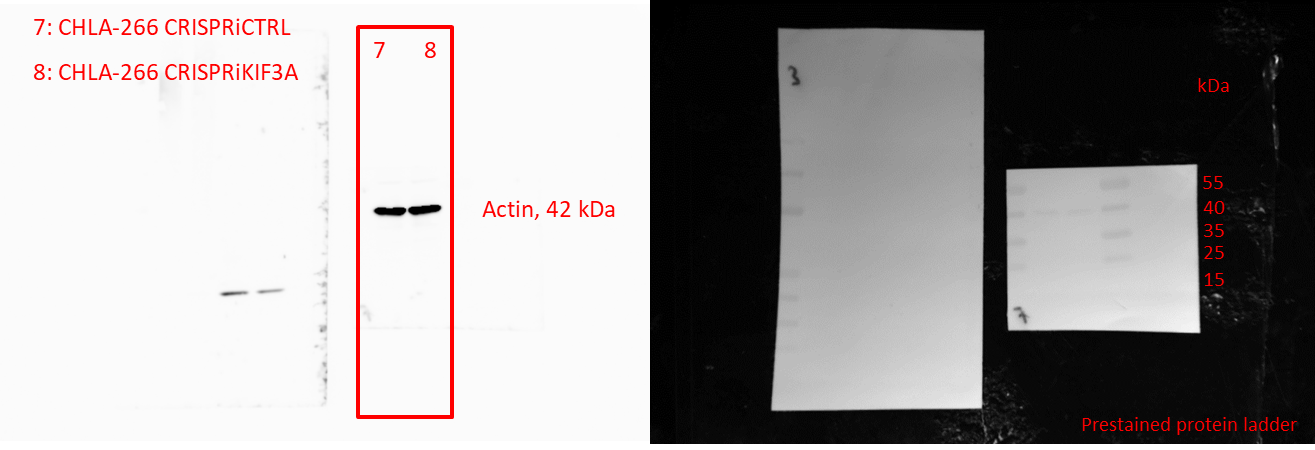
**

Supplement: Supplementary file 10 — Uncropped Western blots [file 41419_2022_5243_MOESM10_ESM.docx]
